# Characterization of the Microbiome at the World’s Largest Potable Water Reuse Facility

**DOI:** 10.3389/fmicb.2018.02435

**Published:** 2018-10-26

**Authors:** Blake W. Stamps, Menu B. Leddy, Megan H. Plumlee, Nur A. Hasan, Rita R. Colwell, John R. Spear

**Affiliations:** ^1^Department of Civil and Environmental Engineering, Colorado School of Mines, Golden, CO, United States; ^2^Department of Research and Development, Orange County Water District, Fountain Valley, CA, United States; ^3^CosmosID, Inc., Rockville, MD, United States; ^4^Department of Cell Biology and Molecular Genetics, University of Maryland, College Park, MD, United States

**Keywords:** water reuse, pathogens, water purification, metatranscriptomics, metagenomics

## Abstract

Conventional water resources are not sufficient in many regions to meet the needs of growing populations. Due to cyclical weather cycles, drought, and climate change, water stress has increased worldwide including in Southern California, which serves as a model for regions that integrate reuse of wastewater for both potable and non-potable use. The Orange County Water District (OCWD) Advanced Water Purification Facility (AWPF) is a highly engineered system designed to treat and produce up to 100 million gallons per day (MGD) of purified water from a municipal wastewater source for potable reuse. Routine facility microbial water quality analysis is limited to standard indicators at this and similar facilities. Given recent advances in high throughput DNA sequencing techniques, complete microbial profiling of communities in water samples is now possible. By using 16S/18S rRNA gene sequencing, metagenomic and metatranscriptomic sequencing coupled to a highly accurate identification method along with 16S rRNA gene qPCR, we describe a detailed view of the total microbial community throughout the facility. The total bacterial load of the water at stages of the treatment train ranged from 3.02 × 10^6^ copies in source, unchlorinated wastewater feed to 5.49 × 10^1^ copies of 16S rRNA gene/mL after treatment (consisting of microfiltration, reverse osmosis, and ultraviolet/advanced oxidation). Microbial diversity and load decreased by several orders of magnitude after microfiltration and reverse osmosis treatment, falling to almost non-detectable levels that more closely resembled controls of molecular grade laboratory water than the biomass detected in the source water. The presence of antibiotic resistance genes and viruses was also greatly reduced. Overall, system design performance was achieved, and comprehensive microbial community analysis was found to enable a more complete characterization of the water/wastewater microbial signature.

## Introduction

Without clean drinking water, human society would not exist as it does today. Yet, many societies face water scarcity due to increasing population growth and land use. Many communities also face a decrease in water quality as demonstrated by increased eutrophication related to agriculture to feed global population growth (Moss, 2011). Within the Southern California region of the United States, all of these pressures have been brought to bear and have resulted in increased water stress. Accordingly, projects that integrate water reuse via the engineered treatment of wastewater have become more desirable across California, the United States, and the world (Wade Miller, 2006). Reuse of municipal wastewater instead of discharge to surface waters augments the water supply of communities reliably, safely, and economically. Planned water reuse is being implemented more than in any time in our history to help meet the needs of growing societies (Jimenez and Asano, 2008). While inherent reuse is a natural part of the earth’s water cycle, human intervention via new technologies, engineering, and knowledge speeds up this process, making it possible to produce highly purified water directly from wastewater for potable purposes.

Given the low-quality source, water reuse can bring public concern over the safety of the produced potable water and requires system engineering and monitoring to provide chemical and pathogen control. Even premise plumbing used for potable water distribution is occasionally associated with pathogenic microorganisms if not carefully monitored or maintained (Williams et al., 2004; Pinto et al., 2012; Potgieter et al., 2018). Regulatory standards for potable reuse vary by state in the United States and worldwide; in California, facilities must use a treatment train achieving 12, 10, 10-log_10_ of virus, *Giardia*, and *Cryptosporidium*, respectively, from the raw wastewater source through to the finished water. This type of approach coupled with regulatory-required routine monitoring of microbial indicators and operational surrogates have been used for many decades to ensure the safety of conventional drinking water as well as recycled water. Diagnostically monitoring the removal of bacterial and eukaryotic pathogens within wastewater remains a process rooted in traditional methods such as coliform plate counts (Fu et al., 2010), yet more modern methods such as DNA sequencing may detect other potentially pathogenic microorganisms that fail to grow using traditional cultivation media (Staley and Konopka, 1985; Santos et al., 2009). Sequencing of RNA, in addition to DNA, may also aide in the detection of active microorganisms in wastewater or biomass digestion systems (De Vrieze et al., 2018). Moreover, high-throughput DNA or RNA sequencing approaches may allow for much more sensitive detection of a broad range of pathogens as evidenced by recent work that investigated biofilms at the same potable water reuse facility as the present study (Leddy et al., 2017). Microbial water quality and community analysis methods were summarized in a recent review of high-throughput sequencing for potable reuse (Leddy et al., 2018). Overall, water reuse is recognized as a sustainable, predictable (and thus reliable) supply of water that is necessary in water-limited regions as part of a diverse water supply portfolio. Thus, it is essential to better understand the microbial load, microbial community, and the presence of mobile genetic elements such as antibiotic resistance genes (ARGs) associated with potable reuse, which can be used to more fully characterize water quality and demonstrate treatment performance.

Mobile ARGs lead to an increase in antimicrobial resistant microorganisms worldwide, and their increasing prevalence in hospitals is of concern as we continue to search for replacement antimicrobial therapies (Boucher et al., 2009). Any water treatment or distribution system should attempt to decrease transmission of ARGs, which are of emerging concern in wastewater treatment (Pei et al., 2012; Pruden et al., 2013). Recently, researchers have found that reclaimed non-potable wastewater harbors a greater number of ARGs than non-reused potable water (Garner et al., 2018) and that critically, these gene clusters are distributed across a broad range of microorganisms rather than any specific group (Hultman et al., 2018). Other work has also suggested that conventionally treated wastewater may actually increase the number of ARGs present within the solid, or biofilm, associated phase of the wastewater stream (Quach-Cu et al., 2018). Removal of ARGs from the water stream prior to potable reuse is clearly preferable and is a topic of current study. Treatment by either microfiltration or ultraviolet (UV) light are proven to be effective (Chang et al., 2017), yet less is known about the efficacy of other technologies and multibarrier, or combined, treatment-train systems integrated with UV.

While potable reuse is broadly accepted and considered safe, there is nevertheless considerable interest in determining what microorganisms (including potential pathogens) are present within a treated wastewater reuse stream and how this changes during treatment. One such water purification and reuse facility is the Orange County Water District (OCWD) Advanced Water Purification Facility (AWPF). The AWPF is a multi-barrier treatment system designed to produce up to 100 million gallons of purified water daily, which is used for local recharge of the region’s groundwater aquifer that is the primary drinking water supply, also referred to as the OCWD Groundwater Replenishment System (GWRS). Previous microbial community analysis (metagenomics) work at the OCWD AWPF has focused on identifying what microorganisms are present in biofilms found on the feed side of filtration membranes including microfiltration (MF, nominal pore size 0.2 μm) and reverse osmosis (RO) membranes as an exploratory proof-of-concept study (Leddy et al., 2017). To date, there has been no comprehensive microbial community analysis to understand the microbial and ARG load in water collected across treatment stages at the AWPF, or similar potable reuse systems.

The goal of this work was therefore to better understand the microbial character and diversity throughout this state-of-the-art potable water reuse treatment facility. The research sought to develop a better understanding of the removal of microorganisms, including pathogens, to advance, inform and optimize multi-barrier water treatment systems. To this end, the microbial community composition at various points in the system were characterized using a combination of small subunit ribosomal RNA (SSU rRNA) gene sequencing, metagenomics, and metatranscriptomics. Metagenome and metatranscriptomic samples were characterized using a novel identification system recently developed (Hasan et al., 2014) to identify microorganisms present throughout the treatment system, as well as viruses and ARGs. Quantitative PCR (qPCR) was used as a complimentary approach to community characterization to provide an estimate of microbial biomass, and to better understand at what points in the treatment train the bulk of microbial removal occurred. Through this concerted approach we have provided the first in-depth characterization of water at a multibarrier potable reuse facility, from influent to final product water.

## Materials and Methods

### Sampling and Nucleic Acid Extraction

**FIGURE 1:**
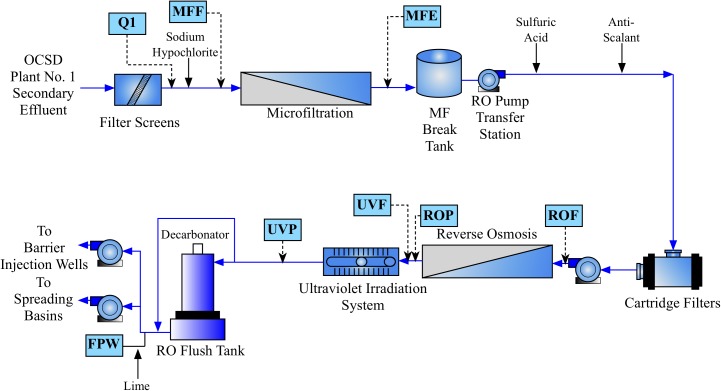
Process flow diagram of the Orange County Water District (OCWD) Groundwater Replenishment System (GWRS) Advanced Water Purification Facility (AWPF). Sample locations are shown in blue boxes, with approximate location in the flow diagram shown by dashed arrows. Hydrogen peroxide is also added prior to UV treatment (not shown).

Water was sampled across multiple points of the AWPF GWRS representing key steps in the treatment process (Figure 1). Specifically, we sampled the plant influent (Q1; secondary-treated, non-disinfected wastewater that is approximately a blend, 80:20 of nitrified activated sludge and trickling filter effluents) treated by the Orange County Sanitation District; microfiltration (MF, nominal pore size 0.2 μm) influent or feed (chloraminated, nominal chlorine residual of 3.5 mg/L as Cl_2_); MF effluent (MFE); reverse osmosis (RO, spiral-wound, thin-film composite polyamide operated at 150 – 200 psi) Feed (ROF); RO permeate (ROP); ultraviolet advanced oxidation process (UV/AOP) feed (UVF); UV/AOP product (UVP which received an estimated UV fluence of 800-900 mJ/cm^2^ and a nominal H_2_O_2_ concentration of 3 mg/L); and finished product water (FPW; stabilized via partial decarbonation and lime addition) that were taken in October of 2016 and July of 2017. The number of sampling events was based on budget, the availability of sampling personnel, and the high stability of the system operation during the monitored period.

All water samples were filtered using the Innovaprep Large Volume Concentrator (LVC) dialysis filter cartridge system (Innova Prep LLC, Drexel, MO, United States). This filter-cartridge based system allowed for the filtration of 100 L of water in less than 1 h per sample location. Briefly, water was fed through the LVC, and measured in 20 L intervals. Once 60 – 100 L of water were processed per sample site, biomass and particulate were eluted off the filter membrane using the supplied sterile phosphate buffered saline (PBS) elution buffer at a final volume of 50 – 100 mL and retained in sterile 250 mL bottles until further processing. Approximately one-fifth of the concentrated volume was retained for microscopy, and another fifth for confirmation of viable microbial populations by cultivation. Further information related to microscopy and cultivation methods can be found in Supplementary Materials. The remaining volume was split evenly and passed through triplicate 25 mm 0.22 μm nitrocellulose filters. Each filter was then placed in a BashingBead^TM^ lysis tube (Zymo Inc., Irvine, CA, United States) containing 750 μL of DNA/RNA Shield (Zymo, Inc.) to immediately preserve samples on-site and limit bias due to sample shipment. After the completion of filtration, all samples were vortexed on-site to disperse the preservation solution evenly before return to the laboratory. All samples for DNA or RNA extraction were maintained at -80°C prior to further processing.

Additionally, biofilm samples were collected from the MF and RO membranes during the July 2017 sampling event to compliment water samples taken from these locations. Briefly, samples of a recently removed MF membrane were excised using a sterile razor blade. The membrane was in operation for approximately 9 years, and fibers were cut from the top, middle, and bottom of the unit. RO biofilm samples were obtained from two units, one in operation for 8 months and another in operation for 1.5 years. Biofilms were obtained by cutting open the RO membrane and then scraping material into a sterile tube using a sterile razor blade. All biofilm samples were immediately frozen at -80°C. Because of the assumed low-biomass nature of the AWPF, filter and DNA extraction control (blank) samples were also prepared to compare to the microbial communities identified within the AWPF. Both DNA and RNA were co-extracted from all samples using the ZymoBIOMICS DNA/RNA Miniprep kit (Zymo, Inc.) according to manufacturer’s instructions. Both purified DNA and RNA were eluted into 100 μL of nuclease free water after extraction. Filter volumes, total extractable DNA, and other summary sample statistics are available in Supplementary Table S1.

### Analysis of Water Quality

Both chemical and microbial water quality are measured daily, weekly, or monthly by OCWD staff (depending on the constituent) for indicator parameters, including anions and cations. To characterize the present study’s water samples taken in October 2016 and verify expected treatment performance, major anions were measured using a Dionex ICS-90 ion chromatography system running an AS14A (4 × 250 mm) column. Major cations were also measured using a Perkin-Elmer Optima 5300 DV Inductively Coupled Plasma Optical Emission Spectrometer (ICP-OES); both IC and ICP were completed at the Colorado School of Mines. Water was filtered at the time of sampling using an 0.22 μm nitrocellulose filter. All ICP samples were acidified with trace-metal grade nitric acid as per standard procedure to ensure mobilization of all metal cations. Continuous online measurements of TOC, turbidity, temperature, and pH were also collected by the AWPF. TOC measurements were made with a Sievers M5310 online TOC Analyzer (SUEZ Water Technologies & Solutions, Trevos, PA, United States). Turbidity was measured with a Hach 1720E (Hach Co., Loveland, CO, United States) and temperature and pH were measured using Rosemount online meters (Emerson Electric Company, St. Louis, MO, United States). The samplers run continuously, and measurements were recorded every 6 h for the period coinciding with the present study from October 2016 to July 2017.

### Molecular Analyses of Water and Biofilm From the AWPF

#### Small Subunit rRNA Gene Amplification

Libraries of bacterial, archaeal, and eukaryotic small sub-unit (SSU) rRNA gene fragments were amplified from each DNA extraction using PCR with primers (Integrated DNA Technologies Co., Coralville, IA, United States) that spanned the ribosomal RNA gene V4 hypervariable region between position 515 and 926 (*Escherichia coli* numbering) that produced a ∼400 bp fragment for bacteria and archaea, and a 600 bp fragment for the eukaryotes. These primers evenly represent a broad distribution of all three domains of life (Parada et al., 2016). The forward primer 515F-Y (**GTA AAA CGA CGG CCA G** CCG TGY CAG CMG CCG CGG TAA-3′) contains the M13 forward primer (in bold) fused to the ssuRNA gene specific forward primer (underlined) while the reverse primer 926R (5′-CCG YCA ATT YMT TTR AGT TT-3′) was unmodified from Parada et al. (2016). 5 PRIME HOT master mix (Quanta BioSciences Inc., Gaithersburg, MD, United States) was used for all DNA reactions at a final volume of 50 μL. Briefly, samples underwent an initial denaturation at 94°C for 2 min, followed by 30 cycles of: denaturation at 94°C for 45 s, annealing at 50°C for 45 s, extension at 68°C for 60 s, followed by a final denaturation at 68°C for 5 min. Reverse-transcription PCR (RT-PCR) was performed for all RNA samples using qScript XLT 1-Step RT-PCR Kit (Quanta Biosciences Inc.). Briefly, RT was carried out at 48°C for 20 min, followed by an initial denaturation of 94°C for 3 min. Otherwise cycling occurred as above. All reactions were purified using AmpureXP paramagnetic beads (Beckman Coulter Inc., Indianapolis, IN, United States) at a final concentration of 0.8 x v/v. After purification, 4 μL of PCR product was used in a barcoding reaction to attach a unique 12 bp barcode to each library in duplicate 50 μL reactions. A mock community was also used as a positive control (Zymo Inc.). Duplicate reactions were pooled, purified using AmpureXP beads to a final volume of 40 μL, quantified using the QuBit HS DS DNA assay kit (Thermo Fisher Scientific Inc., Waltham, MA, United States), and pooled in equimolar amounts before concentration using an Amicon 30 K centrifugation column (Merck Millipore) to a final volume of 80 μL. To mitigate the effects of reagent contamination (Salter et al., 2014) multiple extraction blanks and negative controls were sequenced. The pooled, prepared library was then submitted for sequencing on an Illumina MiSeq (Illumina Inc., San Diego, CA, United States) using V2 PE250 chemistry at the Duke Center For Genomic And Computational Biology.

#### Quantitative PCR

Total bacterial/archaeal small sub-unit (SSU) rRNA gene count was estimated using a TaqMan based probe assay previously designed to provide even and accurate amplification of bacteria and archaea within a sample (Liu et al., 2012). Briefly, the assay was carried out in 25 μL reactions containing 1x final concentration of PerfeCTa qPCR ToughMix (Quanta BioSciences Inc.), 1.8 μM of each primer, and the probe at a final concentration of 225 nM using samples collected and extracted in July 2017 as template for each reaction. Each biological replicate was also assayed in technical triplicate. A seven-point standard curve was generated using serially diluted genomic *E. coli* DNA in triplicate.

#### SSU rRNA Gene Analysis

Sequence reads were de-multiplexed in QIIME version 1.9.1 (Caporaso et al., 2010b). Sequence reads were first denoised and then clustered into sub-operational taxonomic units (sOTUs) at 100 percent identity using UPARSE (Edgar, 2013, 2016). After clustering, sOTUs were assigned taxonomy using mothur (Schloss et al., 2009) against the SILVA database (r132, Pruesse et al., 2007). Each OTU was then aligned against the SILVA r132 database using pyNAST (Caporaso et al., 2010a), filtered to remove uninformative bases, and then a tree was created using the maximum likelihood method and the Jukes Cantor evolutionary model within FastTree 2 (Price et al., 2010). A BIOM formatted file (McDonald et al., 2012) was then produced for use in analyses downstream. To limit OTUs originating from contaminating microorganisms found in extraction and PCR reagents (Salter et al., 2014) all extraction blanks and PCR controls were processed separately, and a core microbiome was computed. Any sOTU found in 75 percent of controls was filtered from the overall dataset. Differences in community composition were estimated using an unweighted UniFrac index (Lozupone and Knight, 2005). Taxa heatmaps and ordination plots were generated using phyloseq (McMurdie and Holmes, 2013) and AmpVis. Prior to analysis in R, all computation was carried out on the XSEDE Jetstream cluster (Towns et al., 2014).

#### Metagenomic/Transcriptomic Sequencing

Extracted DNA was provided to CosmosID (Rockville, MD, United States) after quantification using the Qubit HS or BR assay (Thermo Fisher Scientific Inc., Waltham, MA, United States). Total RNA was converted to complementary DNA (cDNA) using the Protoscript II reverse transcriptase (New England Biolabs, Ipswich, MA, United States). Second-strand cDNA synthesis was carried out using the NEBNext^®^ Ultra II Non-Directional RNA Second Strand Synthesis module (New England Biolabs, Ipswich, MA, United States), and final cDNA samples were quantified using the Qubit HS assay. Ribodepletion was not attempted due to the expected complex mixture of bacteria, archaea, and eukaryotes, and total cDNA was submitted for sequencing at CosmosID. Each DNA sample was normalized in 3–18 μL of nuclease-free water for a final concentration of 0.5 ng/μL using a Biomek FX liquid handler (Beckman Coulter Life Sciences, Brea, CA, United States) prior to library preparation. Libraries were constructed using the Nextera XT Library Prep Kit (Illumina, San Diego, CA, United States), followed by 13 cycles of PCR amplification using Nextera i7 and i5 index primers and 2X KAPA master mix in a modified Nextera XT protocol. The PCR products were purified using 1.0X speed beads and eluted in 15 μL of nuclease-free water and quantified by PicoGreen fluorometric assay (100X final dilution). The libraries were pooled by adding an equimolar ratio of each based on concentration determined by PicoGreen and loaded onto a high sensitivity (HS) chip run on the Caliper LabChipGX (Perkin Elmer, Waltham, MA, United States) for size estimation. Pooled libraries were then sequenced using an Illumina HiSeq 3000 flowcell using PE150 chemistry at CosmosID Inc.

#### Characterization of Unassembled Metagenomic Sequencing Reads

Unassembled metagenomic sequencing reads were directly analyzed using the CosmosID bioinformatics software package and the GenBook^®^ database which includes over 150,000 bacteria, viruses, fungi and protists genomes and over 5,500 antibiotic resistance and virulence associated genes (CosmosID Inc., Rockville, MD, United States). Briefly, the CosmosID pipeline employs two k-mer based algorithms, 5VCE and NmerCE (Hasan et al., 2014), that rapidly identifies unaligned short metagenomic reads against a manually curated microbial reference database and a reference phylogenetic tree (with each species within the reference tree having a unique k-mer “fingerprint”). The approach employed by CosmosID allows for the putative identification of microorganisms at the species or strain level by the classification of individual sequencing reads (Hasan et al., 2014). ARGs were also identified in unassembled metagenomic sequence reads by CosmosID within the GenBook^®^ database using the same approach as above. Identified sequence reads were quantified by relative abundance, or detection based on a threshold of coverage across a target genome. Once generated, analyses were visualized within the CosmosID website and figures generated. Additional information in the methodology employed by CosmosID and the technical aspects of the fingerprinting method are described in detail elsewhere (Hasan et al., 2014; Lax et al., 2014; Ponnusamy et al., 2016).

## Results

### Online and Chemical Water Quality Measurements

**FIGURE 2:**
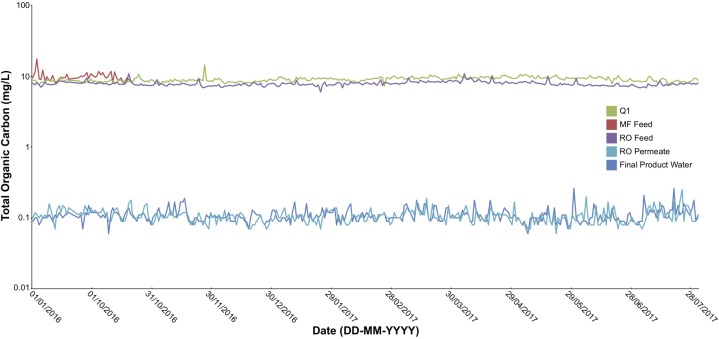
Total organic carbon (TOC) at Q1, MF feed, RO feed, RO permeate, and finished product water sampling points in the AWPF. Measurements taken once every 6 h using online instrumentation across the indicated sample period.

Several key operational parameters were obtained from online readings every 6 h through the AWPF including: total organic carbon (TOC, Figure 2), turbidity, temperature, pH, and total chlorine from September 1, 2016 to July 31, 2017 (Supplementary Figures S1–S4). Turbidity declined from an average of 3.6 Nephelometric turbidity units (NTU) at Q1 (secondary wastewater) to 1.6 NTU at MFF, to 0.08 – 0.04 NTU at ROF, ROP, and FPW. Temperature was stable across the system during the sampling period measured, averaging near 80 F (≈26.7 C), ranging from a low of 79.5 F at ROF to a maximum average of 80.8 F at FPW. pH averaged 7.1 at MFF and fell to 6.9 at ROF, declining to 5.5 to 5.6 at ROP and UVP (as a result of the purification process), before increasing to an average of 8.5 at FPW due to addition of lime coupled with partial decarbonation (for corrosion control of finished water delivery pipelines) before the FPW exited the AWPF. Total chlorine declined throughout the treatment process after initial treatment at MF Feed (Supplementary Figure S3). Similar to other online readings, TOC was stable across the sampling period (Figure 2), declining dramatically with treatment, but stable at each sample point over time. All major anions and cations declined to near detection limits (Supplementary Table S1), with a minor increase in nitrate from ROP to UVP (Table 1). All sampled ions and online measurements agree with historical data from the sample site (Orange County Water District [OCWD], 2016) and demonstrate expected performance of the facility.

**Table 1:** Major anions and cations from water samples taken in October 2016 across the AWPF.

	DL (μM)	Q1	MFE	ROF	ROP	UVF	UVP	FPW
Al	76.05	0.95	0.98	1.37	BDL	BDL	BDL	BDL
B	424.62	33.92	18.93	38.62	24.66	20.82	28.93	23.42
Ba	0.08	0.26	0.14	0.25	0.01	0.02	0.01	0.02
Ca	15.89	1952.52	1106.25	1913.09	2.15	1.47	2.35	318.59
Cd	0.10	BDL	BDL	BDL	BDL	0.00	0.00	0.00
Co	0.42	0.01	0.01	0.01	BDL	BDL	BDL	BDL
Cr	0.65	BDL	0.01	0.01	BDL	BDL	BDL	BDL
Cu	1.72	0.03	0.04	0.13	BDL	BDL	BDL	0.03
Fe	0.57	3.06	1.31	1.78	BDL	BDL	BDL	BDL
K	82.56	423.13	166.12	437.40	16.35	14.28	14.63	17.54
Li	19.89	3.38	1.78	3.37	0.27	BDL	0.26	0.36
Mg	0.52	960.01	573.69	939.28	1.05	0.79	0.88	1.45
Mn	0.07	0.83	0.49	0.72	0.00	0.01	0.02	0.05
Na	585.17	7694.77	7673.89	7723.48	352.37	296.96	311.58	336.25
Ni	0.86	0.07	0.05	0.09	BDL	BDL	BDL	BDL
P	34.15	12.69	8.13	51.72	BDL	1.68	BDL	0.53
Pb	0.96	BDL	BDL	BDL	BDL	BDL	0.01	BDL
S	86.03	1868.04	3512.53	1951.34	11.37	6.20	4.54	18.17
Se	12.68	0.41	0.26	0.64	0.61	0.26	0.23	0.41
Si	40.43	341.29	192.45	339.83	15.94	11.95	26.18	15.27
Sr	0.12	5.64	3.16	5.50	0.01	0.01	0.01	0.07
V	1.63	0.02	BDL	0.02	BDL	BDL	BDL	BDL
Zn	0.46	0.58	0.45	0.72	0.03	0.06	0.08	0.13
Ti	0.34	BDL	BDL	BDL	BDL	0.01	0.00	BDL
F^-^	5.26	42.57	21.58	36.38	BDL	BDL	BDL	BDL
Cl^-^	2.82	7807.46	4169.11	7786.13	229.49	226.97	243.34	224.92
NO2^-^	2.17	9.58	BDL	BDL	BDL	BDL	BDL	BDL
Br^-^	1.25	4.34	2.07	3.38	BDL	BDL	BDL	BDL
NO_3_^-^	1.61	924.41	1699.12	743.41	122.89	437.61	104.67	123.14
PO_4_^3-^	5.26	BDL	BDL	7.58	BDL	BDL	BDL	BDL
SO_4_^2-^	1.04	1928.78	2985.08	2052.05	3.26	18.90	3.33	4.03

*All values are reported in micromolar (μM). BDL, below detection limit*.

### Identification of AWPF Microbial Community

**FIGURE 3:**
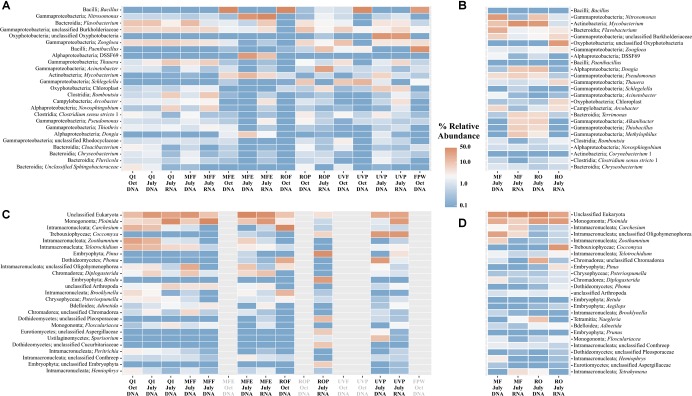
Taxonomy summary from rRNA gene sequence data of the bacteria/archaea in water samples **(A)** or biofilms **(C)**, or eukaryotes in water **(B)** or biofilms **(D)** across the AWPF. Sample locations in gray failed QC, or did not produce sufficient quantities of DNA sequence to process within the eukaryotes. The top 25 genera by relative abundance for all domains of life are shown. Scale shown is in percent relative abundance from low (blue) to high (red) percentages.

**FIGURE 4:**
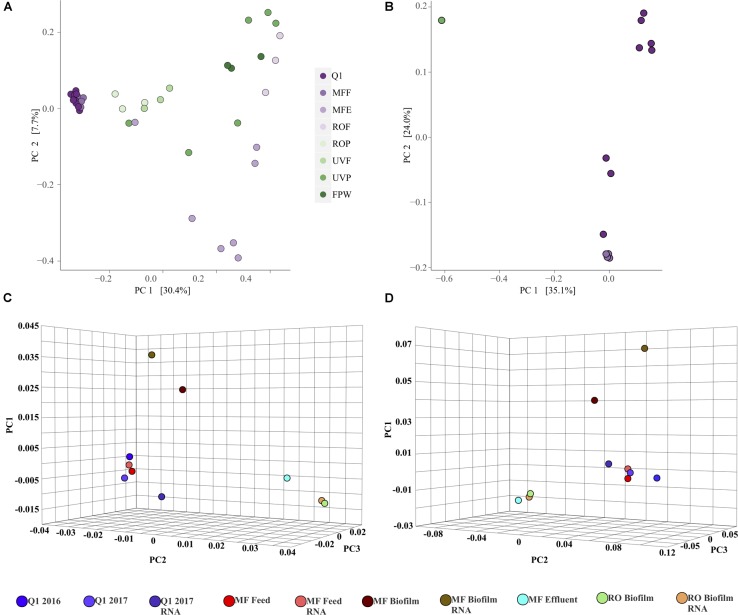
Principal coordinate ordination, produced using an unweighted UniFrac distance matrix for the bacteria/archaea **(A)** or eukaryotes **(B)** from rRNA gene sequence data. Axis values represent percent variance explained by the ordination. Principal component ordination of the bacteria **(C)** or antibiotic resistance genes **(D)** produced using a Bray-Curtis distance matrix within the CosmosID web application. Additional information on the specific types of ARGs used in the ordination of 5d can be found in Supplementary Table S2.

The bacterial community within the AWPF was highly similar in the secondary effluent (Q1 site) and MFF (Figures 3A,C); the difference between these two sites is the addition of sodium hypochlorite to form chloramine for membrane fouling control in the treatment system. Differences between Q1 and MFF were confined to the Clostridia and Betaproteobacteria, with sOTUs most closely related to *Romboutsia* and Clostridium *sensu strictu* group 1 and *Thauera* in greater abundance in RNA samples at Q1. RNA samples at both Q1 and MFF were more similar to one another than DNA samples from each site (Figure 3A). After MF treatment, the MF effluent was variable between the October to July sampling events, with *Bacillus* dominating in October, while both DNA and RNA samples at MFE in July 2017 were dominated by sOTUs most closely related to *Nitrosomonas, Flavobacterium*, DSSF69 (Class Alphaproteobacteria), *Mycobacterium*, and *Dongia.* Samples from ROF and ROP contained a greater number of sOTUs including unclassified Gammaproteobacteria, *Zoogloea, Paenibacillus*, and numerous other genera (Supplementary Table S1). Samples at UVP also contained a high relative abundance of sOTUs most closely related to unclassified Oxyphotobacteria and *Paenibacillus*. Samples differed in community composition from all controls which were dominated by *Escherichia-Shigella, Halomonas, Ca. Alysiosphaera, Achromobacter*, and others (Supplementary Figure S5). By unweighted UniFrac distance matrix ordination, the microbial communities of the AWPF appear to be visually separate pre- and post- RO filtration (Figure 4A). Overall, the microbial communities were significantly distinct between sample locations (*p* = 0.001, *R*^2^ = 0.551). No bacterial cells were identified by scanning electron microscopy (SEM), but thick biofilm-like cellular mass was present in water from Q1, MFF, and in the biofilm of the MF membrane (Supplementary Figures S6–S8). By phase contrast microscopy, multiple bacterial cell morphologies were present in Q1 and MFF water samples (Supplementary Figure S9). Bacterial isolates were recoverable from Q1 and MFE in October 2016, but not at any sample site downstream from MFE (Supplementary Figure S13). These results are consistent with routine AWPF monitoring which indicate the presence of bacteriological indicators in Q1 and MFF samples, but their absence at all downstream sites (data not shown). Additional SEM imaging from the remainder of sampled locations in October 2016 is available in Supplementary Material.

Detectable eukaryotic DNA sequence was only identified at Q1, MFF, MFE, ROF, and ROP. No sequence passed QC for other locations (further along the treatment train). Only Q1, MFF, MFE, and ROP RNA samples produced sequence of sufficient quality to determine relative abundances of the eukarya. Across all samples, unclassified eukaryotes were the most abundant, followed by the class Monogononta, a class of rotifers (Figures 3B,D). Cillates most closely related to the genera *Zoothamnium* and *Telotrochidium* were detected in DNA samples at Q1, but absent in RNA samples at MFF (Figure 3B). As with the bacteria and archaea, sites were significantly different although with a weaker correlation coefficient (*p* = 0.003, *R*^2^= 0.361). Only samples from Q1, MFF, and a single outlying sample from UVP produced sufficient sequence data (≥1000 sequence reads) to be visualized by principal coordinate ordination within the eukaryotes (Figure 4B). Large eukaryotic cells including diatoms and potential rotifers were identified by scanning electron (Supplementary Figures S6–S8) and phase contrast microscopy (Supplementary Figure S9). A summary of per-library statistics for both the bacterial/archaeal, as well as the eukaryotic sequencing libraries is available in Supplementary Table S1.

### Metagenomic and Transcriptomic Sequencing of All Three Domains of Life, Viruses, and ARGs

Water samples from Q1 in October 2016 and Q1, MFF, and MFE in July of 2017 produced sufficient quantities of DNA for metagenomic sequencing. All water samples beyond Q1 in October and beyond MFE in July failed to produce sufficient quantities of DNA needed for sequencing, despite the high filtration volumes employed (Supplementary Table S1). Biofilm samples from MF and RO membranes both generated successful sequencing libraries. A total of 380 million paired-end sequence reads were obtained across all samples. Per-sample summary sequencing statistics can be found in Supplementary Table S1.

**FIGURE 5:**
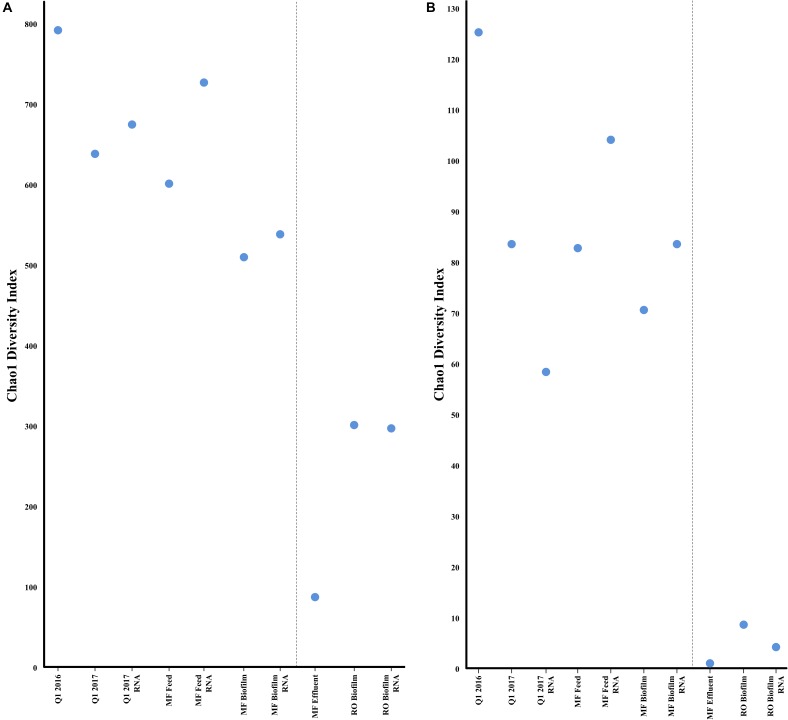
Alpha diversity (Chao1) of metagenomic and transcriptomic samples for bacteria **(A)** or antibiotic resistance genes **(B)** estimated by the CosmosID pipeline. Greater values indicate a greater number of bacterial species, or antibiotic resistance genes.

As expected, bacterial community richness was greatest at Q1 (2016 and 2017) than in other sampled sites. A total of 2,180 bacterial species were identified at Q1 water in 2016, compared to 1,864 species in the 2017 Q1 water sample, correlating to a Chao1 diversity estimate of 790.40 and 637.78, respectively, of bacterial species (Figure 4A). Biofilm samples from MF and RO membranes were less diverse than the Q1 water sample, with species diversity estimated to be 64% lower in the RO and 38% lower in the MF. The lowest estimated bacterial diversity was identified in the MF effluent water (MFE), which was only 11% of the estimated diversity in Q1 water from 2016 (Figure 5A). Similar to the above 16S rRNA gene sequencing analysis, samples appeared to cluster pre- and post- filtration (Figure 4C) with Q1 and MFF clustering more closely together than the remainder of the samples by principal component analysis.

Both fungi and parasites were identified in metagenomic and metatranscriptomic sequence libraries, though far fewer unique species, or sequence reads were assigned to either lineage relative to the Bacteria. The greatest number of fungal species was identified in Q1 water (July 2017 sampling) from DNA/metagenomic and RNA/metatranscriptomes analysis (*n* = 24). A large shift in the relative abundance of fungal species was observed between Q1 water DNA and cDNA samples (Supplementary Figure S14). The least number of fungal species were identified in the RO biofilm samples. *Clavaria fumosa, Enterocytozoon bieneusi, Epichloe sylvatica, Lentinus polychrous, Malassezia globosa, Malassezia restricta, Mitospordium daphnia*, and *Puccinia arachidis* were identified in all samples taken in July 2017. Fewer parasites compared to fungi were identified in all samples (Supplementary Table S1 and Supplementary Figure S15). Specifically, *Acanthamoeba mauritaniensis, Acanthamoeba palestinensis, Hammondia hammondi* strain H and *Paramecium biaurelia* strain V14 were identified in all samples. Again, Q1 water had the greatest number of parasite species (July 2017 sampling, *n* = 38). The fewest parasite species were identified in the MF biofilm RNA sample, suggesting even fewer active parasites in the MF biofilm, possibly due to the activity of sodium hypochlorite on parasites within the biofilm.

A broad swath of ARGs were detected across all samples (Q1, MFF and MFE), including resistance to aminoglycosides, beta-lactams, quinolones, macrolides, tetracycline, trimethoprim, and others, with the greatest diversity identified in Q1 (Supplementary Table S2). A greater diversity of ARGs were identified in the Q1 water 2016 sample than in Q1 water 2017 (Figure 5B). Similar numbers of ARGs were identified in the Q1 water (2016 and 2017) and in MF biofilm ranging from ≈83,000 to ≈ 260,000 sequence reads identified as ARGs prior to the microfiltration membrane. However, far fewer ARGs were identified in the MF effluent water and RO biofilm samples (Supplementary Figure S16A) with a range of ≈ 2,700 to 7,600 sequence reads identified in the MF effluent water and RO biofilm samples. The number of ARGs had no relation to the total number of DNA sequence reads in each sample (Supplementary Figure S16B). Samples clustered similarly by ARG presence as they did for bacterial communities, with Q1 and MFF appearing more similar than other sequenced samples visualized by PCA (Figure 4D). Both the water samples clustered closer to each other than with biofilms at either MF or RO, suggesting that the ARG DNA sequences identified in the water samples are more similar to each other than the ARGs identified in the biofilms.

### Estimation of 16S rRNA Gene Copy Number From AWPF Water

**FIGURE 6:**
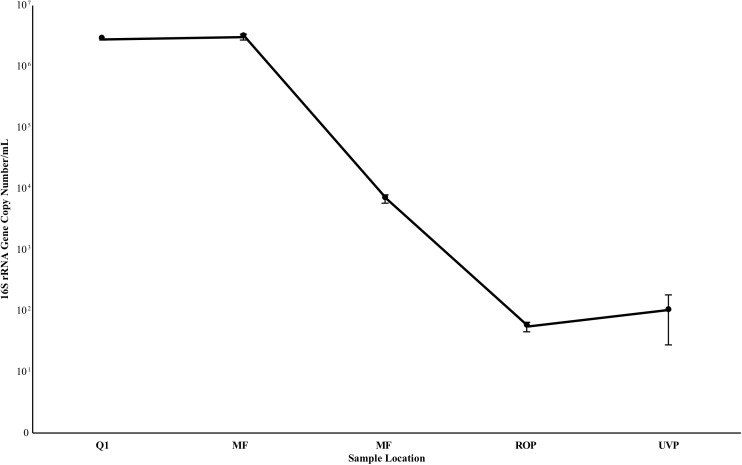
16S rRNA gene copy estimate from samples taken in July 2017. Values are the average of triplicate biological samples and triplicate technical replicates, per biological sample. Reported deviation is the standard deviation of the above samples. Deviation at Q1 was too low to be visualized on the plot. *Y* axis is log scale.

Quantitative PCR results are summarized in Figure 6. Gene copy number estimates remained high at Q1 and MFF ranging from 2.73 – 3.02 × 10^6^ copies 16S/mL (S.D. 1.07 – 3.01 × 10^5^), decreasing by three orders of magnitude from MFF to MFE to 6.78 × 10^3^ (S.D. 1.12 × 10^3^, Figure 6). Negative controls processed in a second qPCR run suggest that any sample below 10^2^ copies 16S rRNA genes/mL was below the limit of detection of the assay indicating that RO and UV/AOP product water were below detectable limits with values of 5.49 × 10^1^ (S.D. 9.87) and 1.02 × 10 ^2^ (S.D. 7.43 × 10^1^), respectively (Figure 6).

## Discussion

Worldwide, communities face water scarcity due to accelerated population growth, land-use issues, short- and long-term drought, climate change and eutrophication of critical water supplies (Madigan et al., 2018). To meet demand, water reuse must escalate and is increasingly advocated and accepted as we seek to reduce the pressure on ever more critical water resources (Wade Miller, 2006). A comprehensive understanding of water quality from treatment, to tap, and throughout the reuse process is therefore paramount. Reusing municipal wastewater that has undergone tertiary or advanced water treatment, with the level of treatment dependent on the application and regulatory requirements, rather than disposal offers municipalities throughout the world a chance to augment their water supply. The OCWD AWPF evaluated in this study is representative of advanced treatment for potable reuse used in many other locations and serves greater than 2.4 million people across Orange County, California. Water chemistry and microbial community were remarkably consistent in this study for the two sampling events, in agreement with historic OCWD water quality weekly profiling (Orange County, 2017).

Prior to MF or RO filtration in the AWPF, the bacterial and archaeal community appears much as expected in secondary treated wastewater, i.e., a reflection of wastewater treatment microbiota. Samples clustered by their location along the treatment process both in the rRNA gene sequence dataset and the CosmosID metagenomic data (Figures 4A,C). This confirms that independent of the method used, broad community structure was similar, although more detailed investigations of the microorganisms present are necessary and needed. Abundant OTUs in the rRNA gene sequencing dataset most closely related to *Flavobacterium*, as well as the Gammaproteobacterium genus *Zoogloea*, and unclassified Comamonadaceae were found in both Q1/MFF, and beyond through ROP and UVP, although not in great relative abundance at MFE. Gammaproteobacteria such as *Zoogloea* are common in activated sludge wastewater treatment systems, are responsible for the bulk of organic carbon removal (Shao et al., 2009; Cydzik-Kwiatkowska and Zielińska, 2016) and their presence is expected. *Flavobacterium* are also common in wastewater and are associated with the formation of flocculent material in activated sludge, which explains their high relative abundance in the influent stream that is comprised of a mix of activated sludge and trickling filter effluent (Tezuka, 1969).

Other abundant microorganisms such as *Thauera* are also common in activated sludge treatment facilities and can cause issues in both dewatering and filamentous sludge bulking (Jiang et al., 2012; Cydzik-Kwiatkowska and Zielińska, 2016). The microbial signal at Q1 and MFF therefore, represents a signal of the co-mingled influent, rather than any specific community unique to the first stages of an advanced treatment facility or this facility itself. Further downstream in the AWPF system, the microbial community began to resemble the microbiota found in conventional drinking water treatment schemes; in general, MF is expected to remove the vast majority of bacteria (and thus wastewater-derived bacteria) and protozoa given the small size of the MF pores (nominal 0.2 μm), while RO provides an even finer size-exclusion removing remaining bacteria as well as viruses. Another possible explanation for the differences in the microbial communities after MF filtration is that the effluent passes briefly (∼20 min) through an equalization tank and then the treatment facility adds chemicals to inhibit RO scaling; thus, the difference between MFE and ROF sites is the brief storage and addition of sulfuric acid and antiscalant. The uncultivated Alphaproteobacterial lineage DSSF69, which was found in a drinking water treatment pilot-scale system previously (Williams et al., 2004) was most abundant at MFE along with *Nitrosomonas*, just after microfiltration. Previous work has shown that members of the Sphingobacteria (including DSSF69) are found in abundance alongside nitrifying bacteria such as *Nitrosomonas* at low chloramine residual levels, which would explain their co-occurrence at MFE (Bal Krishna et al., 2013). *Dongia* and *Mycobacterium* were also abundant at MFE and beyond, both of which are commonly found in water treatment systems and related biofilms (Le Dantec et al., 2002; Liu et al., 2010). Non-tuberculosis mycobacterium can occur within treated wastewater (Amha et al., 2017) although the resolution of the V4-V5 region of the 16S rRNA gene is insufficient to determine their pathogenicity within this study. Other abundant community members included the genus *Bacillus*, but no positive identification within the genus that can contain potentially pathogenic microorganisms was made. Several taxonomic lineages increased in relative abundance in RNA samples including *Romboutsia, Clostridium sensu stricto 1*, and *Thaurea.* Recent work has shown that 16S rRNA can be more representative of the active community than 16S rRNA gene sequence data in flowing or reactor environments (De Vrieze et al., 2018). Our work highlights that there may be differences in the potentially metabolically active microbial community within the AWPF, although additional sampling to unravel the temporal complexity of such a system is needed to fully confirm this assertion. The exact metabolisms of the microorganisms identified by 16S rRNA gene sequencing are beyond the scope of this study but will be the focus of future work. Furthermore, the appearance of high relative abundances of these organisms should not be taken as large numbers of these microorganisms, and additional quantitative measures must be used to establish not only presence, but microbial load.

While rRNA gene sequencing allows for the identification of novel microbiota, or for the characterization of large numbers of samples at low cost and extremely low requirements for input DNA, of greater concern within a wastewater reclamation and treatment system is the detection of potentially pathogenic microorganisms. Metagenomic sequencing is, to some degree, reliant on a well curated database to accurately identify pathogens. Despite the potential limitations of databases in identifying all microorganisms within a sample, metagenomic sequencing has become a solid tool for the rapid identification of pathogens. As recognized, 16S rRNA gene sequencing is a poor choice for the identification of pathogens (Janda and Abbott, 2007), as potential pathogens share high rRNA gene sequence homology with non-pathogenic, or less infectious relatives. Therefore, metagenomic sequencing was employed that enables strain-level resolution and was necessary to distinguish pathogenic from non-pathogenic strains. Q1 water had the greatest diversity and number of bacterial species identified by the CosmosID bioinformatic pipeline, yet no pathogenic microorganisms were detected in abundance in Q1 nor at any sampled point in the treatment process (MFF and MFE). Detected microorganisms by the CosmosID pipeline at Q1, MFF, or the MF and RO membrane sampled biofilms, were more commonly associated with wastewater sludge (Jiang et al., 2012) or human feces (McCarthy et al., 1988; Costello et al., 2009; Toscano et al., 2017) and were well upstream in the AWPF’s treatment process for production of finished water.

Compared to the 100s or 1000s of bacterial species identified, very few fungi or protist species (dozens) were identified at any point in the treatment scheme, despite the large amount of DNA and RNA sequence obtained, suggesting that they represent very little of the total microbial community. Two identified fungi, *Enterocytozoon*, detected in Q1, MFF, MF biofilm, and RO biofilm, and *Onygenales*, detected across all samples, are potential human pathogens infecting immunocompromised individuals (Sulaiman et al., 2014). Despite the identification of these two fungi, overall the fungi represented less than 1 percent of the sequence reads identified in influent (Q1) water, and were otherwise plant pathogens such as *Puccinia* and *Lentinus* (Fangkrathok et al., 2014; Gill et al., 2015). Protists were in similarly low abundance, representing less than 1 percent of the identified metagenomic or metatranscriptomic sequence data. *Paramecium aurelia*, a common environmental non-pathogenic protist (Siegel, 1958), was the most abundant overall. The pathogen *Plasmodium falciparum* (Mehlhorn, 2008) was detected, again representing only a fraction of a percent of the total sequence identified. No metagenomic or transcriptomic sequence was obtained beyond MFE in water samples, due to the low amount of DNA/RNA recovered in spite of large filtration volumes, highlighting the key observation of a reduction in biomass detectable by qPCR at each step in the treatment process within the advanced water/reuse treatment system. Future work is needed to optimize methods to enable metagenomic and transcriptomic analysis of RO permeate, UV/AOP product water, and similarly ultra-pure waters from potable reuse systems, to characterize the community living in this challenging environment, demonstrate treatment performance, and further increase public confidence in the safety of the water.

If the microbial community detected by rRNA gene or metagenomic/transcriptomic sequencing are taken in isolation, it would appear as though many community members reappeared after microfiltration and to some degree after RO, approximating the microbial community found at Q1 in both distribution and relative abundance. Indeed, RO product water had a greater number of sOTUs than the MF effluent water. However, a key point is the vast reduction in total biomass after MF and RO, alongside the expected effectiveness of improving both chemical and microbial water quality via the treatment process (Orange County Water District [OCWD], 2016). Abundance detected by rRNA gene sequencing is relative; that is to say, that the percentages identified in figures relate to only the total number of organisms detected within that sample and does not indicate the total number of microbial cells (microbial density) within that sample. Microbial density, rather than differences in community relative abundance, has been found to be a critical component in disease status in the human microbiome (Vandeputte et al., 2017), and a similar correlation is likely in water purification and distribution systems. It is already known that filtration can impact what organisms are detected during treatment (Pinto et al., 2012) and that these organisms can slough off and reappear in downstream sample points, even if they are not in great absolute abundance. Microbial density, therefore, is a critical component of ecology that cannot be ignored and similarities in microbial community profiles, or a rise in the number of detected taxa in rRNA gene sequence studies, should not be misinterpreted as incomplete water treatment.

Chloramination is the first treatment point in the AWPF and a well-proven method to disinfect drinking water (Aieta and Berg, 1986). However, contact time (CT) is critical for disinfection, and was not represented in the samples taken directly after chlorination at MFF that showed no difference in microbial load or community distribution relative to Q1, which is expected given that the intent of chloramination is limited to membrane biofouling control. The starkest differences in microbial load occurred at the two physical (filtration-based) treatment barriers: MF and RO. MF was highly effective in removing a large percentage of the population. A small percentage of the population is nevertheless retained downstream in the MF effluent water (MFE), likely owing to a combination of: (a) incomplete (not 100%) removal of microbiota given that industrial scale MF is not an absolute, perfect barrier, as evidenced by the need for periodic fiber pinning in the facility based on daily pressure testing; and (b) the expected non-sterile conditions after MFE. Further reductions in biomass occurred via RO, and again, a high percentage of the population was removed despite the similarity of the microorganisms detected by rRNA gene sequencing. This heavy depletion of biomass from the system also explains why no metagenomic sequencing libraries were able to be prepared from any water samples beyond the MFE despite the large quantity of water filtered. The system is highly effective in treating water and removal of biomass. Microfiltration was effective in removing whole cells and transmissible genetic elements such as ARGs– a point observed by the large drop in the diversity and number of detectable ARGs at the MF membrane filtration point.

Not only did the RO membrane further deplete the microbial biomass estimated by 16S rRNA gene qPCR and (as expected) significantly reduce most detectable major ions, but it served as an effective barrier to transmissible genomic elements such as ARGs that may otherwise be transmitted to other organisms in the environment once the water is used or discharged (Pruden et al., 2013). The depletion of most major ions and TOC (Table 1 and Figure 2) decreases the likelihood that any heterotrophic microbiota are actively growing, as also evidenced by the decrease in 16S rRNA gene copy number throughout the system. The MF membrane appears to be highly effective in the depletion of ARGs from the water; the greatest removal of ARGs occurred at the MF membranes, as observed by the sequenced biofilm samples at both MF and RO, and MF effluent relative to other sample points. No ARG data exists for water beyond the MF effluent. Additional sampling extending filtration volumes beyond 100 L to allow for metagenomic sequencing of RO permeate and final product water may allow us to fully confirm the efficacy of ARG removal at the AWPF and other similar systems. Transfer of antibiotic resistance from the environment to human pathogens is of concern and any treatment method, such as the employed MF and RO membranes, should strive to remove this risk as a precautionary approach (Boucher et al., 2009; Pei et al., 2012).

Previous research has shown the effectiveness of membrane-based systems, like MF and RO at the AWPF, in removing antibiotic resistance markers from water (Pruden et al., 2013). Compared to all other samples analyzed by high-throughput sequencing (HTS), MFE had the fewest identifiable known ARGs, and the fewest bacterial species or detectable viral sequence. Of note is the difference in the water and biofilm ARG profiles. Our result that the ARG DNA sequences identified in the water samples are more similar to each other than to the ARGs identified in the biofilms (Figure 4D) could indicate that the resident ARGs in a biofilm community, versus potentially transient ARGs in water, presents a diagnostic difference in system performance microbiota (e.g., biofilms) and the flowing ARGs through a system. This could then lead to questions of ARG commonality, exposure, uptake, or the diagnostic nature of ARGs in general, inherent to any treatment system. Further massive HTS approaches applied to such systems will help to answer such questions. Free-flowing DNA was likely not represented in water samples sequenced throughout the AWPF, while all biofilm samples likely contain both ARGs in live cells as well as any free DNA trapped in the exo-polysaccharide (EPS) matrix. Free flowing DNA may represent a reservoir of ARGs further in the treatment system, particularly in biofilms (Guo et al., 2018), although biofilms in the RO membrane at the AWPF showed a decline in the diversity of identifiable ARGs relative to the sampled MF biofilm (Figure 5B) and total number (Supplementary Table S1). In this AWPF system, DNA quantities were too low to successfully sequence beyond MFE from filtered waters using metagenomic or metatranscriptomic sequencing necessary to identify ARGs, despite filtration of up to 100 L of water at each sample location. Future work could focus on the extraction of free- or environmental DNA that may contain ARGs, rather than a filtration-based approach. The multi-barrier treatment system consisting of MF, RO, and UV/AOP treatment for potable reuse, overall, represents an effective means of removing the microbial population present in secondary treated wastewater, in this way fully removing the wastewater “identity” or signature of the wastewater source.

At present, as with more conventional drinking water treatment, there is no statutory requirement or objective for the OCWD AWPF to entirely remove or inactivate (sterilize) the finished water. An original goal of the work described was to characterize the final treated water using metagenomic sequencing, yet the system was so effective in the removal of microorganisms that we were unable to produce sufficient quantities of DNA for metagenomic sequencing even with large volumes of sampled water. As described previously, in the case of OCWD AWPF, the finished water is injected and percolated into the regional aquifer (groundwater basin) where it commingles with groundwater. Following underground storage and travel times to drinking water production wells on the order of months or years, it is then withdrawn by local cities and water agencies as a drinking water source (Leddy et al., 2017). These agencies provide limited additional treatment required for groundwater supplies, such as disinfection. A potentially interesting topic for future study, related to the highly effective removal of the microbial community by the advanced treatment, are the impacts of the injection of high-purity, finished water, on the native groundwater microbial community (if present) in a subsurface aquifer. Cell numbers in the subsurface average near 10^3^–10^4^ cells per mL (Colman et al., 2017), far lower than the concentration of cells in secondary treated wastewater that is the influent into the AWPF (≈ 10^6^ cells per mL) yet likely much higher than the finished, highly purified water from the AWPF. Thus, the water injected into the subsurface likely dilutes what little native microbial community exists. What—if any—impact this might have on the subsurface microbial community and the ‘groundwater finishing’ of a water should be a focus of future work, which should continue to consider the presence or absence of an active microbial population.

Through a combined DNA/RNA sequencing approach, we were able to identify the resident microbial community present within the waters and biofilms of the OCWD AWPF to provide a better understanding of the microbial load from inflow (secondary treated wastewater) through advanced treatment finishing with UV/AOP-treated water. Metagenomics have been used to study environmental systems, human health topics in medicine, and in other applications; in this study, to the authors’ knowledge, this approach was applied to potable reuse for the first time. Future work will further characterize the metagenomic and metatranscriptomic data obtained from this study, including an assembly binning based approach (Eren et al., 2015) to more clearly understand what Bacteria and/or Archaea are present throughout the AWPF. Furthermore, through sampling and microbial community analysis of advanced treatment systems such as the OCWD AWPF repeatedly over the course of several years, rather than through the two timepoints presented within this research, we may be able to identify new diagnostic bacterial or viral markers for such systems, potentially providing industry value for targeted, routine facility monitoring of potable reuse water. The analysis of ultra-clean water such as that sampled from RO and beyond requires increased sampling volumes. Expanding filtration volumes beyond 100 l, or the use of whole-genome amplification techniques, would allow for analysis of RO and other highly purified waters that was unable to be assessed as a part of this work. Finally, additional correlation to other water quality indicators such as online measurements and compliance parameters at reuse facilities may identify useful relationships between specific microbial community members and functional performance of the treatment system. The holistic approach used herein provides a high-resolution view of the microbial communities within a well-functioning advanced water purification facility during normal operation. Without question, the employed multi-barrier treatment approach at the OCWD AWPF is successful in removing the bulk of detectable biomass, as well as potentially harmful ARGs from the built environment. Such a system seeks to solve some of the demands placed on global water use that are only increasing.

## Data Availability Statement

Raw SSU rRNA gene amplicon data can be found in the NCBI SRA under the accession SRR7234393 and SRR7234394. Metagenomic and metatranscriptomic sequencing data can be downloaded from the NCBI SRA under the accessions SRR6439730 – SRR6439740. Scripts used to generate 16S and 18S rRNA gene sequencing analyses and figures are found at the Zenodo accession 10.5281/zenodo.1414505.

## Author Contributions

JS, ML, and RC proposed the work and provided the initial experimental design. BS completed field sampling and laboratory work with assistance from ML. BS led the writing of the manuscript and all authors contributed to writing and editing of the manuscript.

## Conflict of Interest Statement

RC is the owner of CosmosID, Inc. and NH is employed by CosmosID, Inc. The remaining authors declare that the research was conducted in the absence of any commercial or financial relationships that could be construed as a potential conflict of interest.
