# Supplementary material for: Characterization of the Microbiome at the World’s Largest Potable Water Reuse Facility
Source: Front Microbiol. 2018 Oct 26;9:2435. doi: 10.3389/fmicb.2018.02435 (PMC6212505; doi:10.3389/fmicb.2018.02435)
Supplement: Supplementary file 3 [file Data_Sheet_1.docx]

Supplementary Material

Characterization of the Microbiome at the World’s Largest Potable Water Reuse Facility

**Blake W. Stamps^1^, Menu B. Leddy^2^, Megan H. Plumlee^2^, Nur A. Hasan^4^, Rita R. Colwell^3,4^, John R. Spear^1*^**

^1^Department of Civil and Environmental Engineering, Colorado School of Mines, Golden, Colorado, USA

^2^Research & Development Department, Orange County Water District, Fountain Valley, California, USA

^3^Department of Cell Biology and Molecular Genetics, University of Maryland, College Park, Maryland, USA

^4^Cosmos ID, Inc., Rockville, Maryland, USA

*** Correspondence:**John R. Spear
jspear@mines.edu

# Supplementary Methods

- 1. **Scanning Electron Microscopy and Confirmation of Viable Microbial Populations**

A single filter from each sample site was taken for scanning electron microscopy (SEM) and another for the determination of the presence of viable cells. Filter papers were imaged on a Hitachi TM-1000 scanning electron microscope (Hitachi High-Technologies America, Inc., Schaumburg, IL) under environmental conditions with no fixation. Individual filter papers were affixed to the microscope stage with carbon tape and allowed to dry under vacuum prior to imaging. Each filter was imaged under multiple magnification levels, and the majority of the surface of each sample was scanned to identify any possible microbial morphology present.

Aerobic cultivation was attempted on 1/10^th^ strength tryptic soy agar (TSA) supplemented with 4.5 g/L NaCl to maintain osmotic balance within the medium, and R_2_A medium_._ Briefly, filter papers were immersed in phosphate buffered saline and vortexed to remove any biomass. Approximately 100 µL was spread onto both media types for each sampled location in October 2016 and allowed to grow until a visible lawn was present, or no growth was observed after ≈ 2 weeks at room temperature. Subsequently, unique colony morphotypes were chosen, and struck successively a minimum of three times until isolation was achieved.

# Supplementary Results

- 1. **Scanning Electron Microscopy and Viable Growth**

Scanning electron and phase contrast microscopy images obtained from October 2016 and July 2017 samples are shown in figures S6-S9, and S10-S12. Diatoms were visible at Q1 (Figure S6), in addition to apparent microbial biofilm. Little to no particulate was present on filters obtained post microfiltration (Figures S10-S12). Distinct bacterial morphology was difficult to identify by SEM, although cells were heavily embedded in extracellular polymeric substance (EPS) in samples taken from Q1 at both sampling events. Subsequent phase contrast microscopy from Q1 and MFF showed a dense microbial population, with likely bacterial and eukaryotic morphotypes (Figure S9). Cultivation produced viable growth in October 2016 samples from Q1 and MFE only, with representative morphotypes shown in figure S13. All isolates were preserved on 25 % glycerol, and stored at -80 ˚C. No further characterization was attempted beyond microscopy.

# Supplementary Figures and Tables

## Supplementary Figures


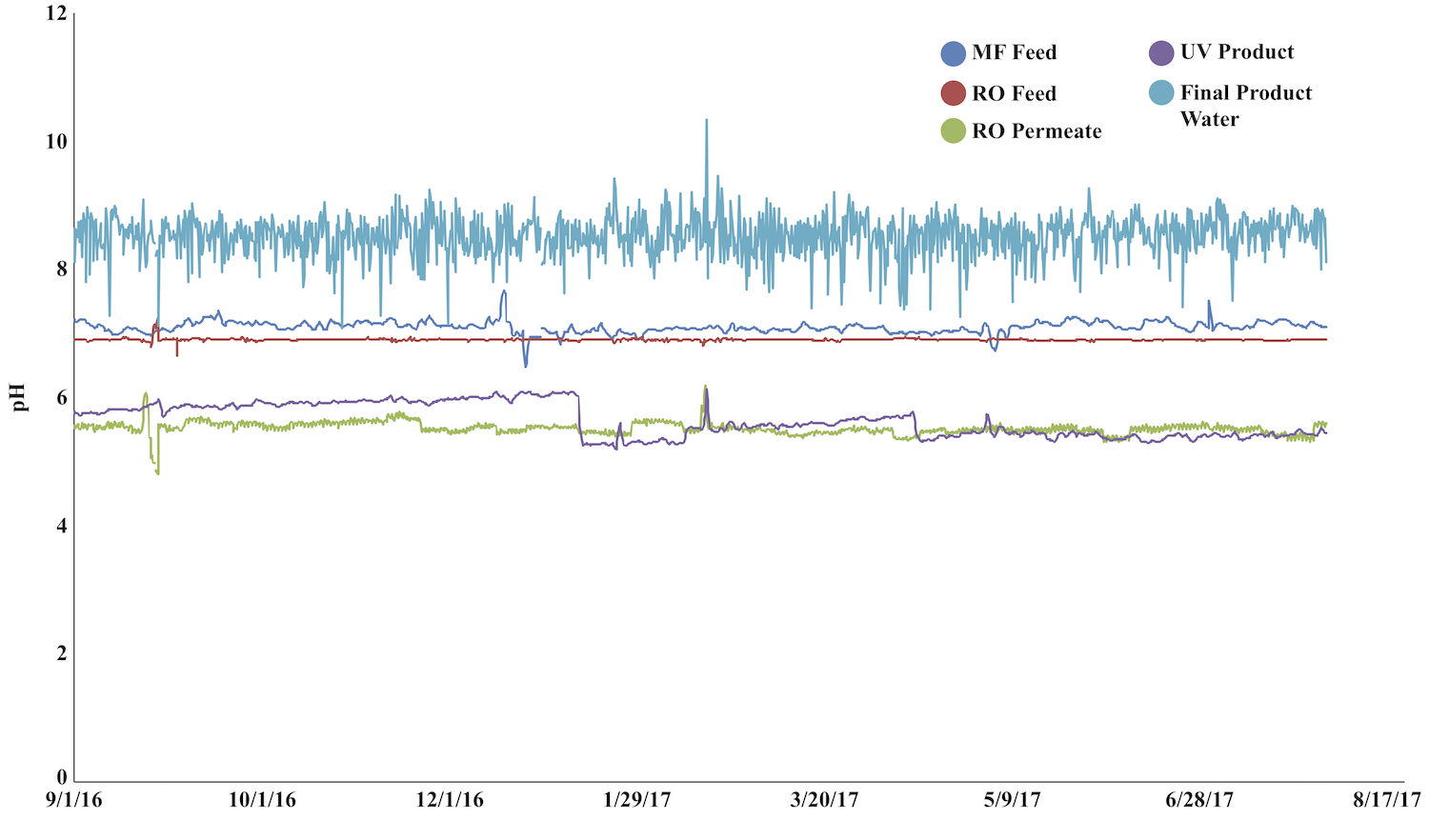


**Supplementary Figure 1.** Continuous pH measurements taken online at MF Feed, RO Feed, RO permeate, UV product, and final product water (FPW).


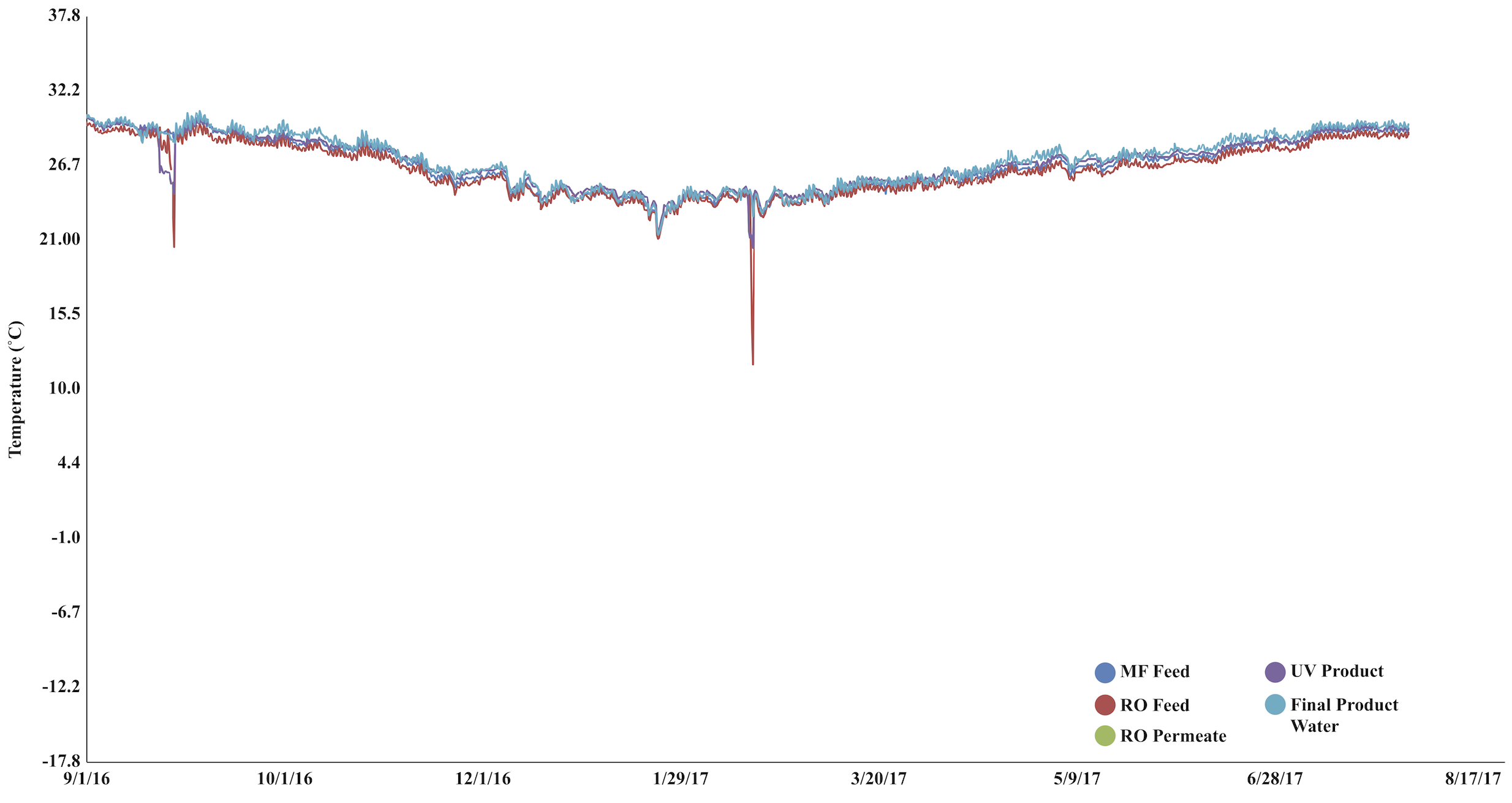


**Supplementary Figure 2.** Continuous temperature measurements taken online at MF Feed, RO Feed, RO permeate, UV product, and final product water (FPW).


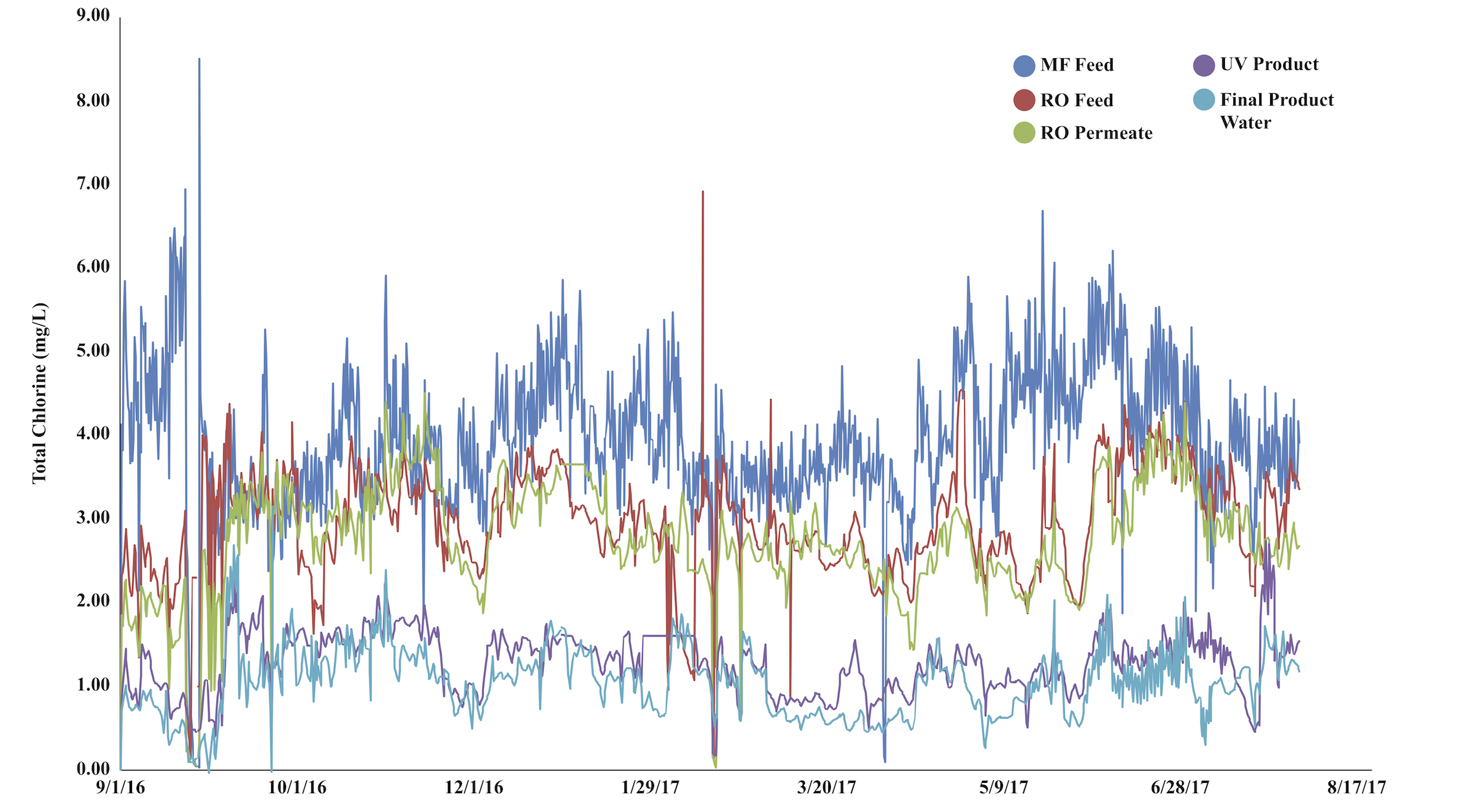


**Supplementary Figure 3.** Continuous total chlorine measurements taken online at MF Feed, RO Feed, RO permeate, UV product, and final product water (FPW).


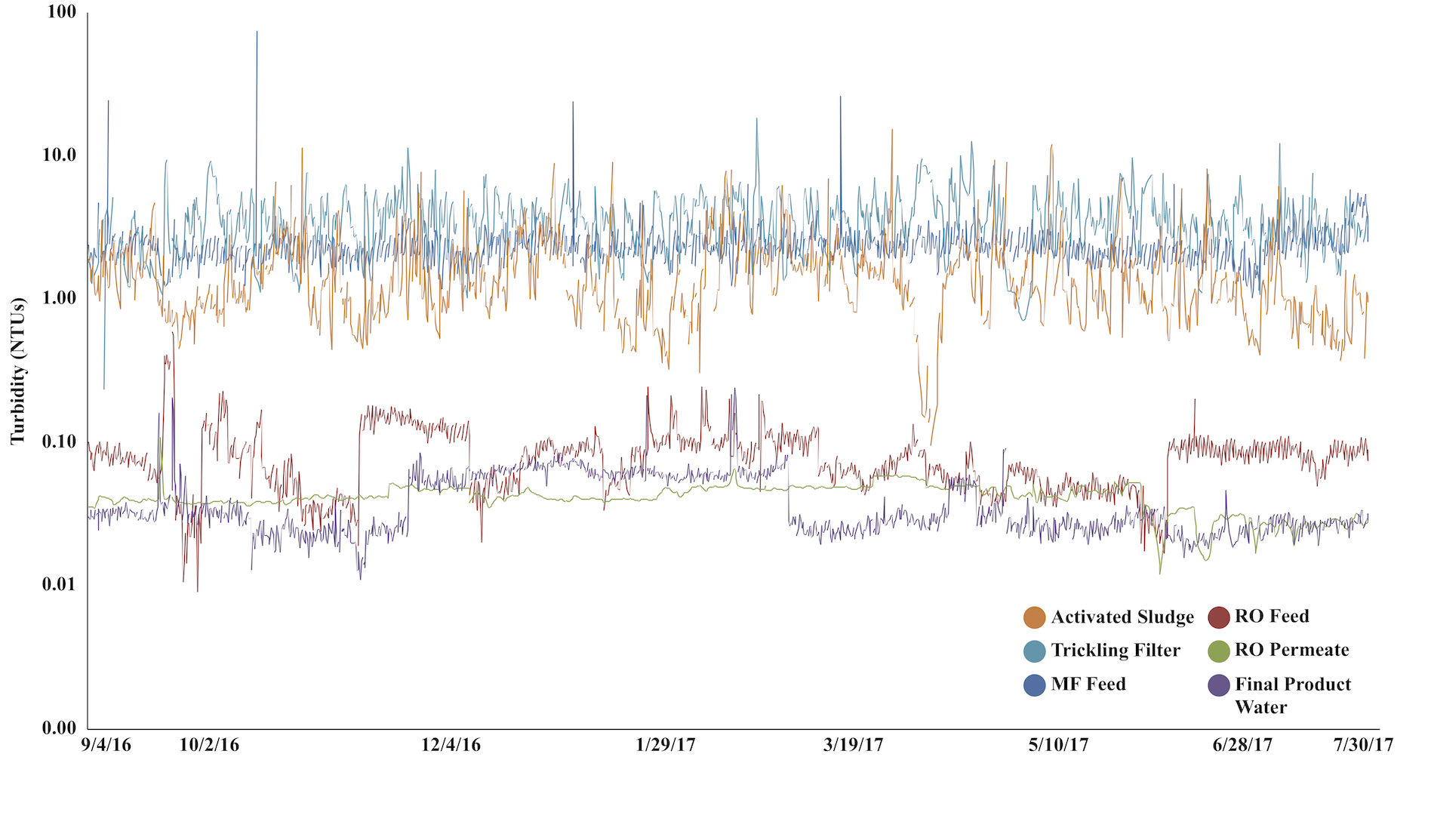


**Supplementary Figure 4.** Continuous turbidity measurements taken online at trickling filter and activated sludge effluent (representing the two input streams for the AWPF), MF feed, RO feed, RO permeate, and final product water (FPW).

**Supplementary Figure 5.** 16S/18S rRNA gene sequenced controls from both sequencing runs in October 2016, and July 2017 of the bacteria/archaea (A) and the eukaryotes (B). Mock community sequenced purchased from Zymo, Inc.


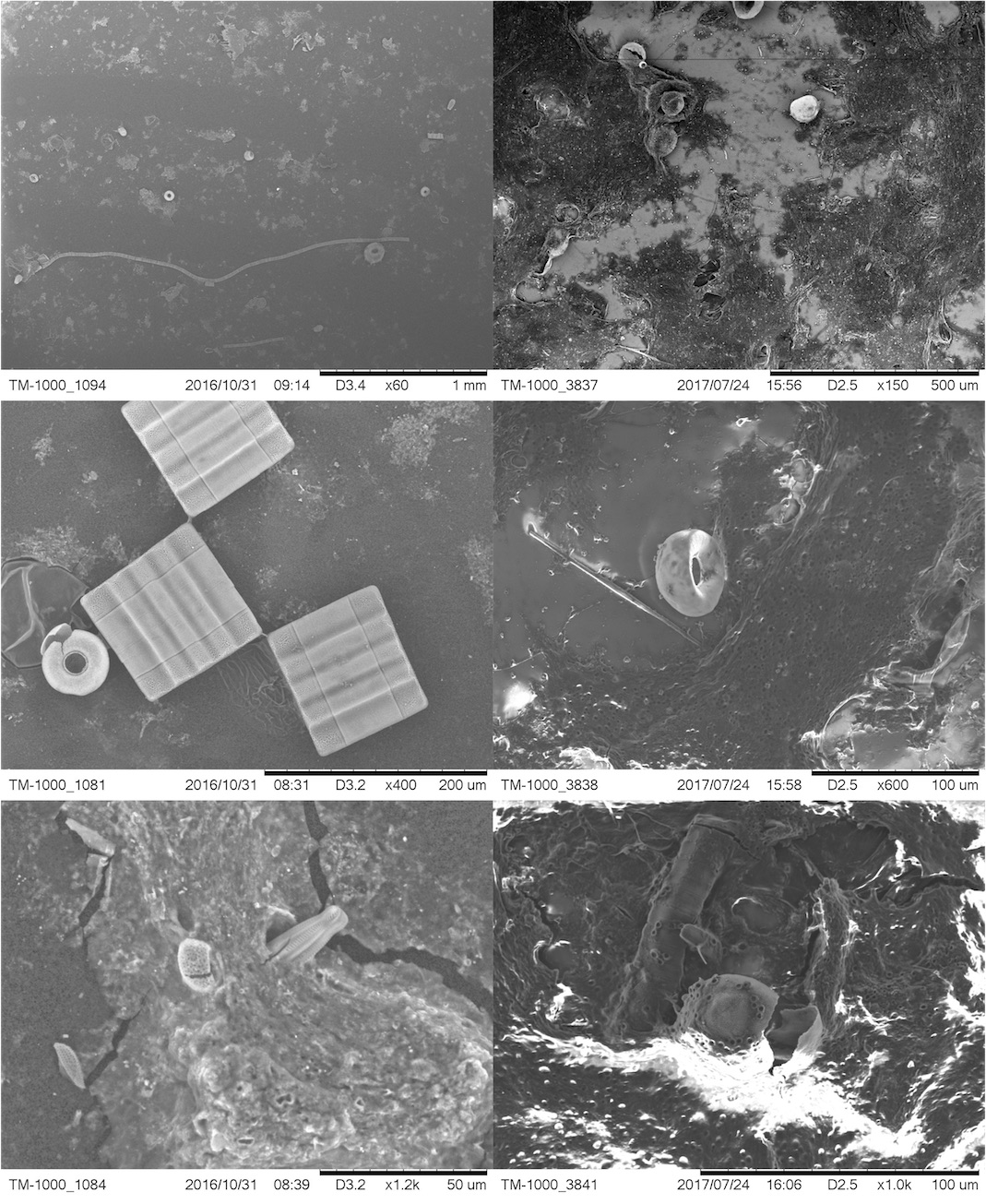


**Supplementary Figure 6.** Scanning electron micrographs from Q1 taken in October 2016 (Left) and July 2017 (Right).


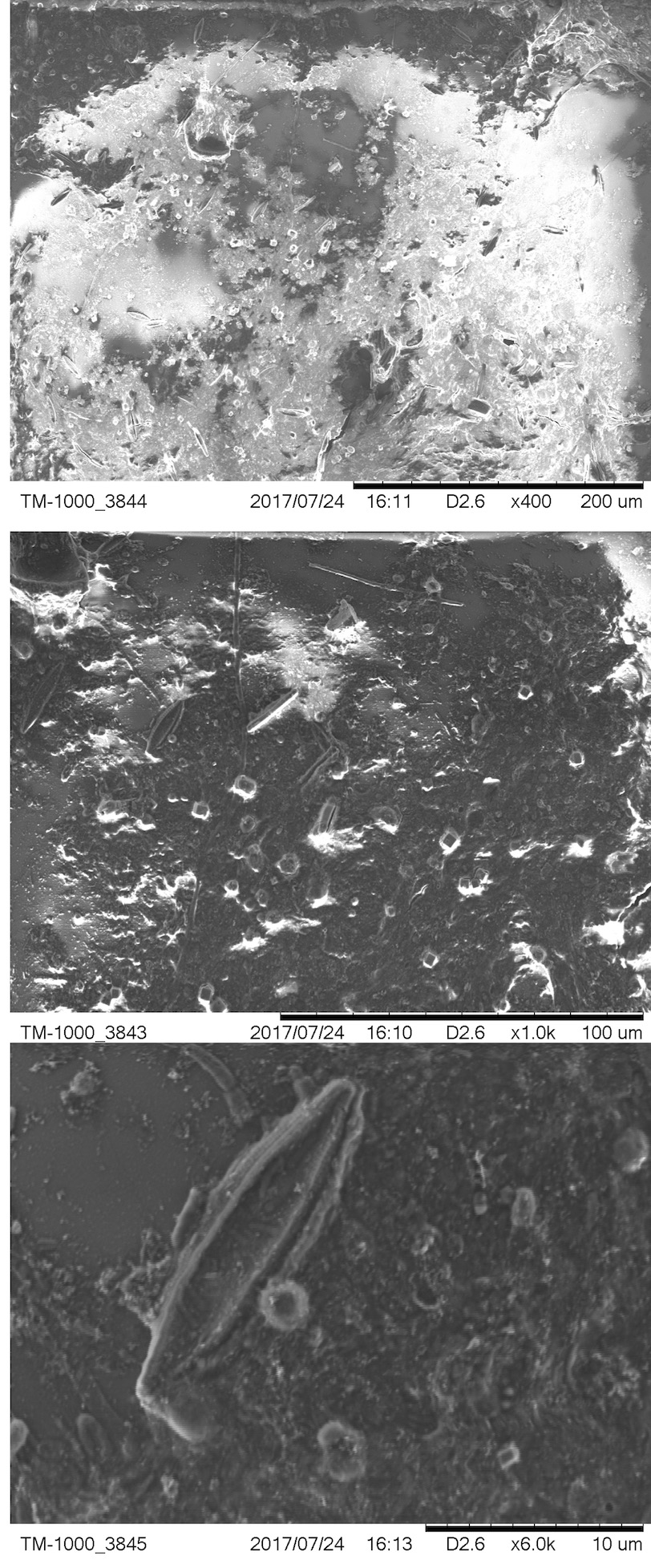


**Supplementary Figure 7.** Scanning electron micrographs from MFF taken in July 2017 .


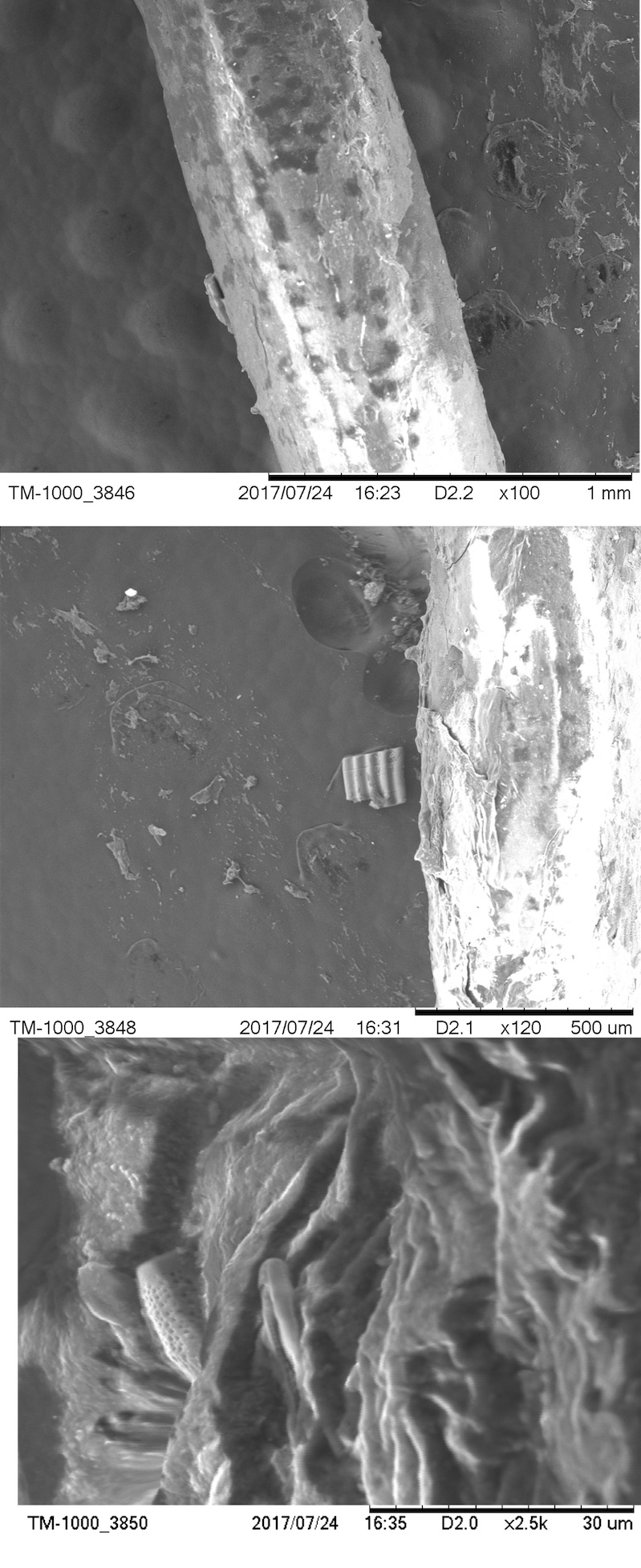


**Supplementary Figure 8.** Scanning electron micrographs from the MF membrane taken in July 2017.


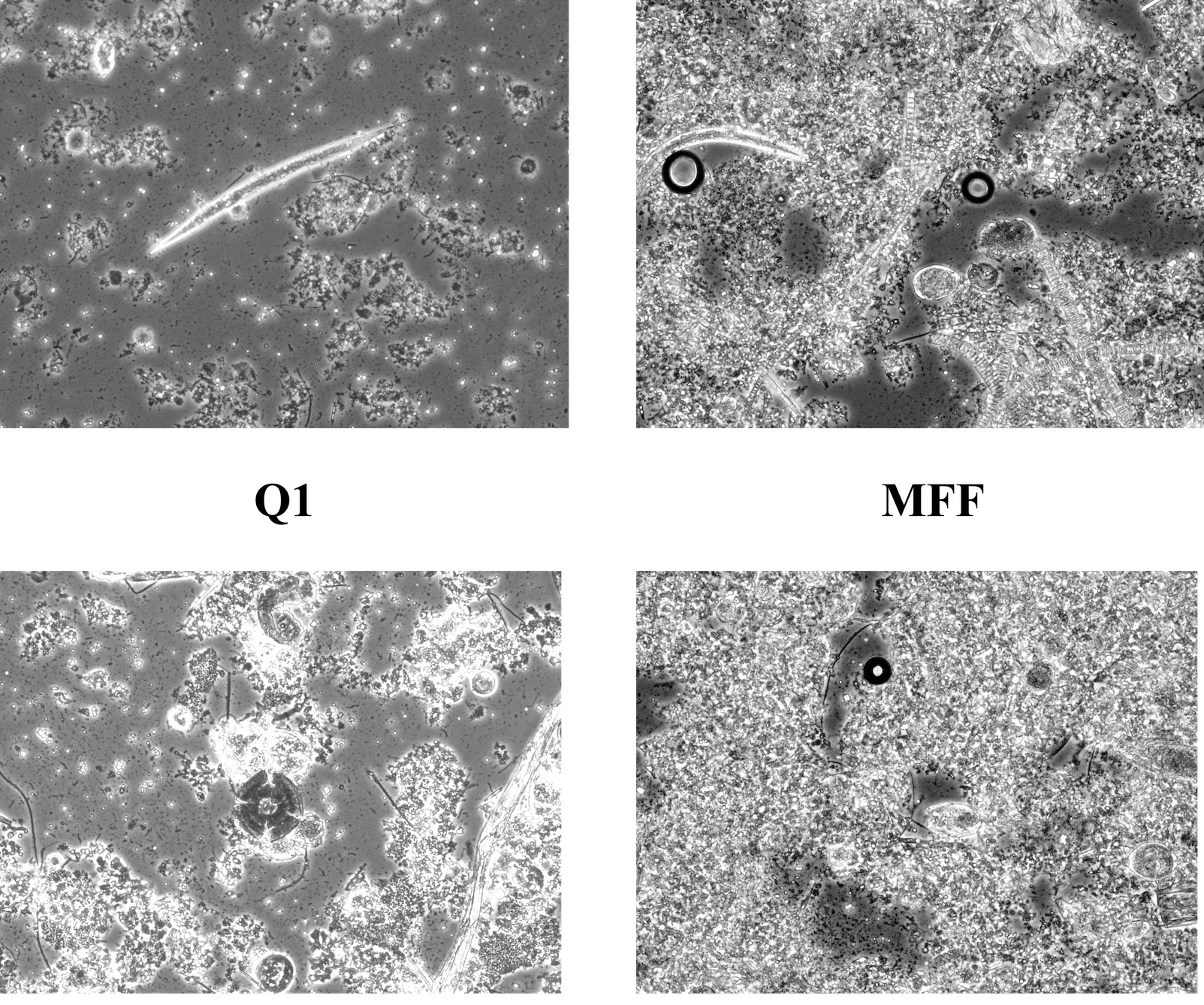


**Supplementary Figure 9.** Phase contrast microscopy from Q1 (Left) and MFF (Right) in July 2017. Images taken at 40 x objective, 10 x ocular magnification.


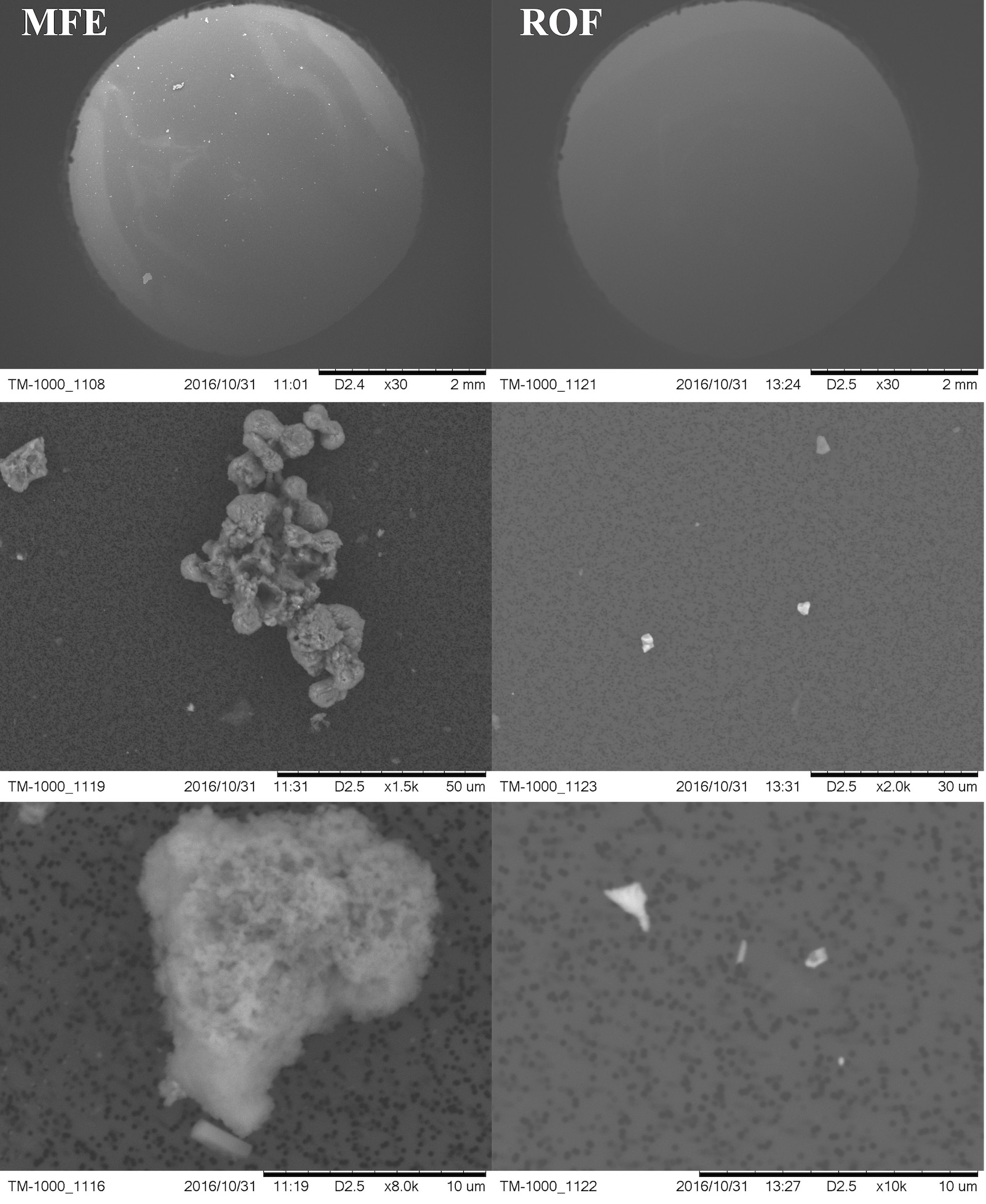


**Supplementary Figure 10.** Scanning electron micrographs from MF effluent (Left) and RO feed (Right) water samples membrane taken in October 2016.


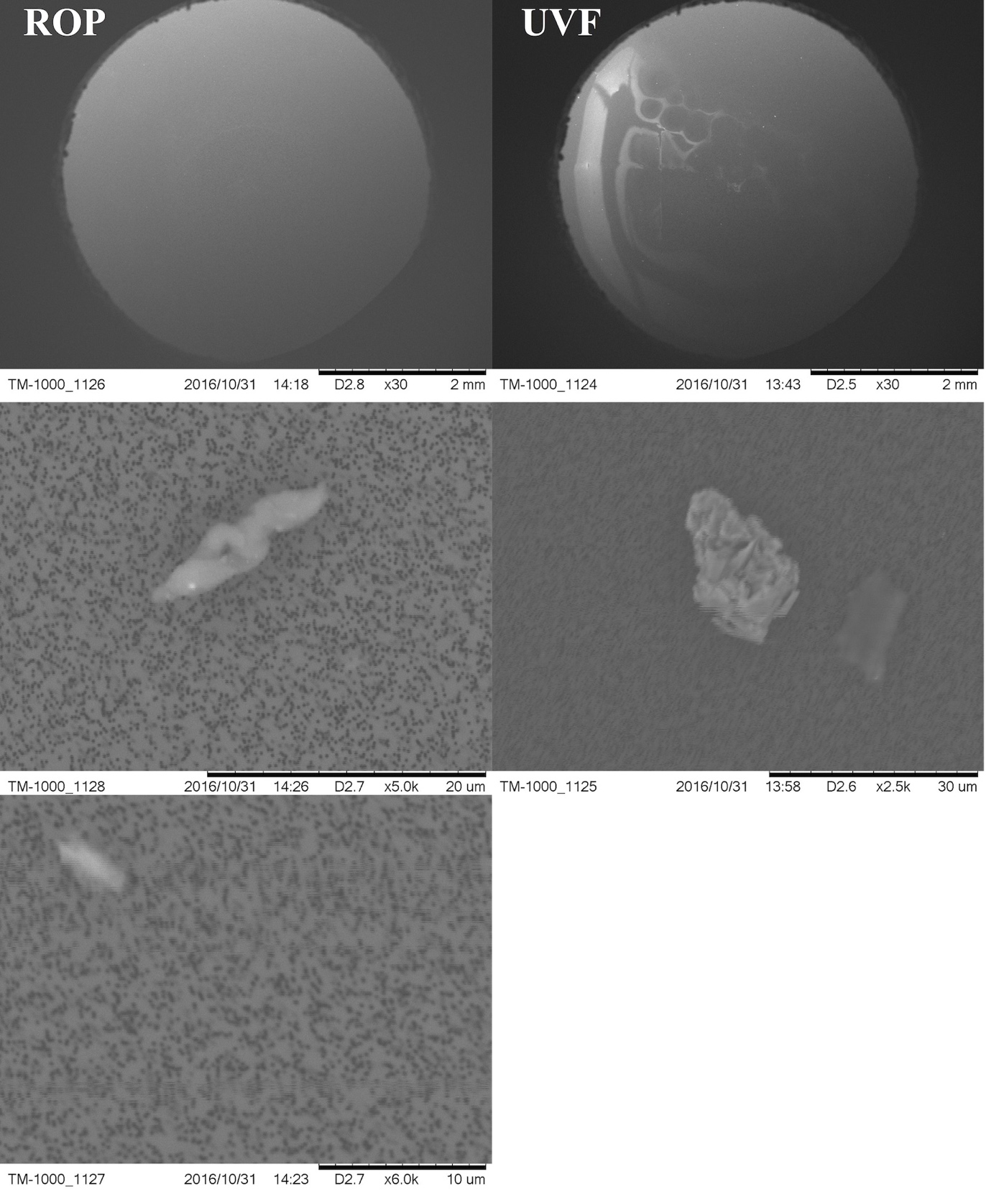


**Supplementary Figure 11.** Scanning electron micrographs from RO permeate (Left) and UV feed (Right) water samples membrane taken in October 2016.


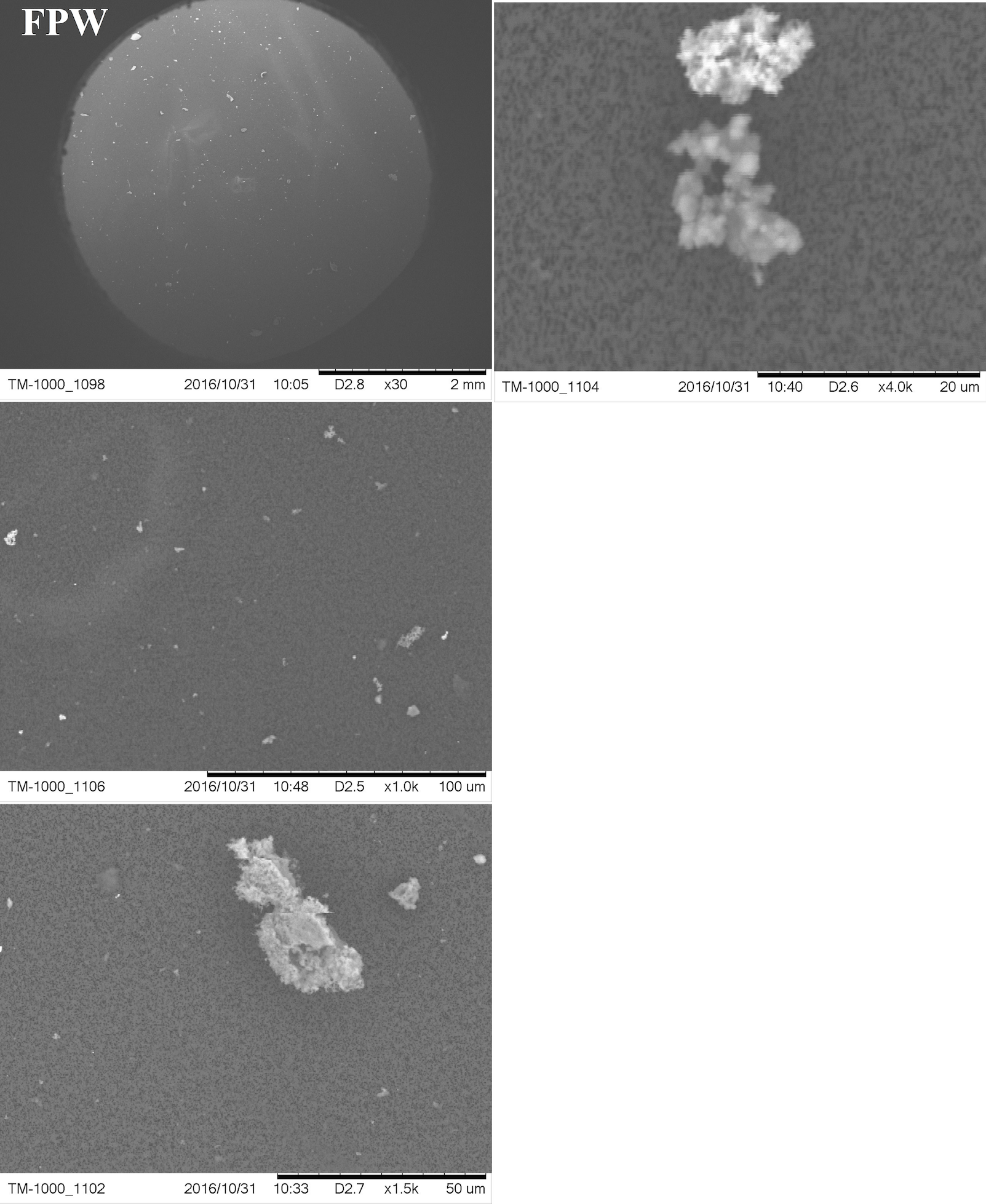


**Supplementary Figure 12.** Scanning electron micrographs from final product water sample membrane taken in October 2016.


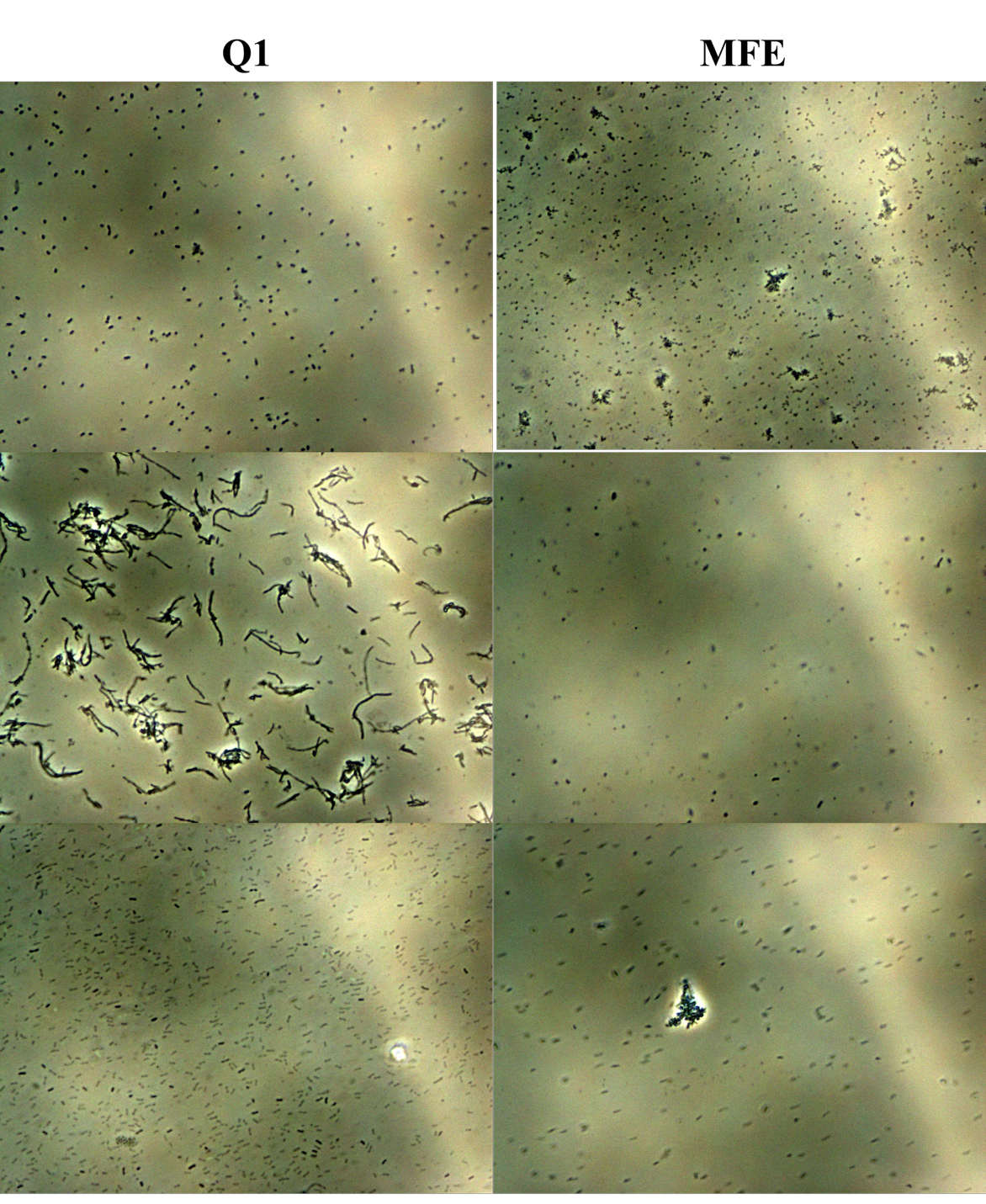


**Supplementary Figure 13.** Phase contrast microscopy from isolated microorganisms at Q1 and MFE, from environmental samples taken in October 2016. Sample images obtained at 40 x objective, 10 x ocular magnification.

**Supplementary Figure 14.** Summary of detected fungi, by percent relative abundance of each sample. A table is also avalible as supplemental table S2.

**Supplementary Figure 15.** Summary of detected protists, by percent relative abundance of each sample. A table is also avalible as supplemental table S2.


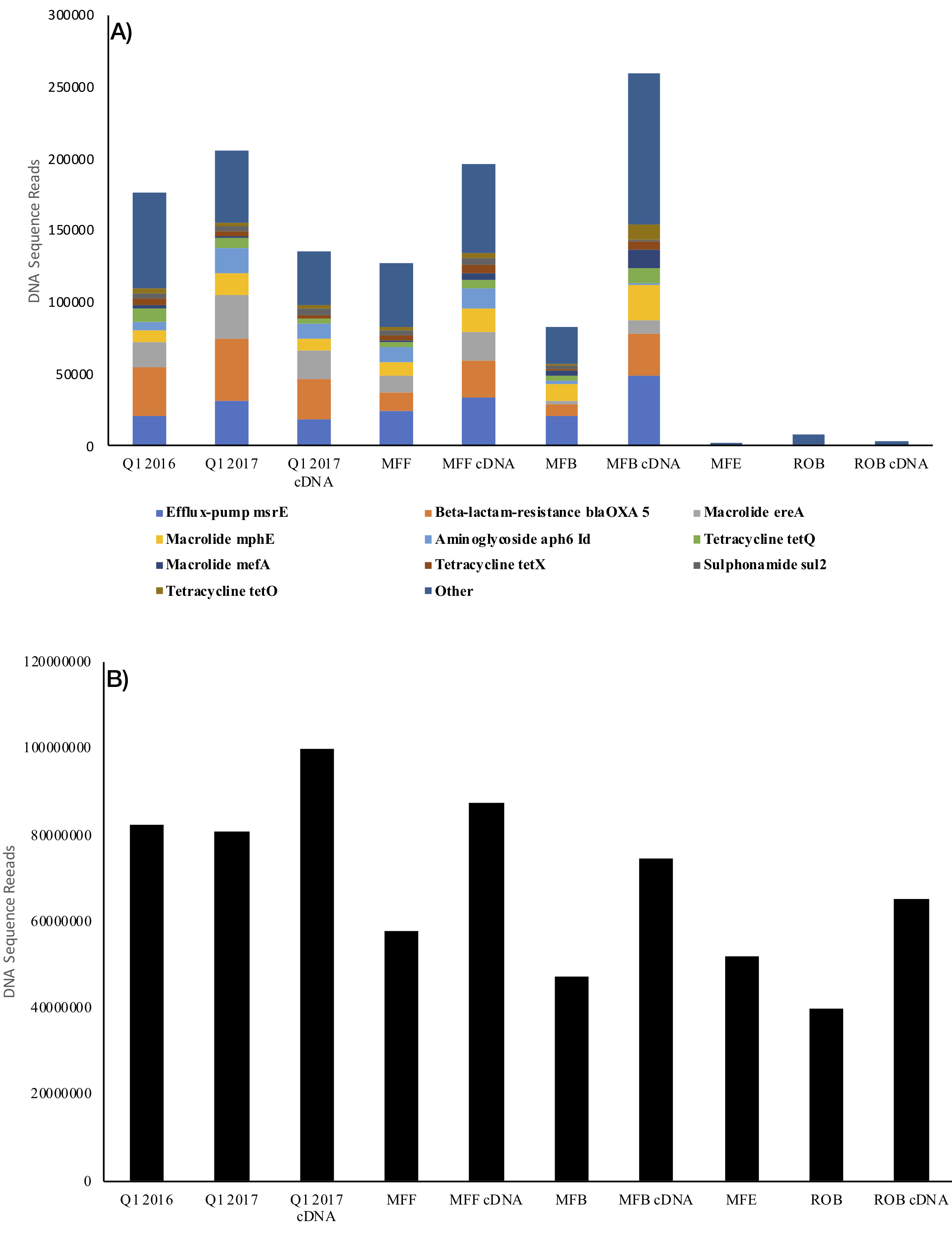


**Supplementary Figure 16.** Summary of antimicrobial resistance genes (ARGs) detected using the CosmosID platform. The ten most abundant ARGs, shown as the total number of gene sequence reads (A) and the total number of DNA sequence reads (B) for each sample.
